# A Resealed-Cell System for Analyzing Pathogenic Intracellular Events: Perturbation of Endocytic Pathways under Diabetic Conditions

**DOI:** 10.1371/journal.pone.0044127

**Published:** 2012-08-29

**Authors:** Fumi Kano, Daiki Nakatsu, Yoshiyuki Noguchi, Akitsugu Yamamoto, Masayuki Murata

**Affiliations:** 1 Department of Life Sciences, Graduate School of Arts and Sciences, The University of Tokyo, Meguro-ku, Tokyo, Japan; 2 PRESTO, Japan Science and Technology Agency, Saitama, Japan; 3 Department of Cell Biology, Nagahama Institute of Bio-Science and Technology, Nagahama, Shiga, Japan; Institut Curie, France

## Abstract

Cell-based assay systems that can serve as cellular models of aberrant function in pathogenic organs would be novel and useful tools for screening drugs and clarifying the molecular mechanisms of various diseases. We constructed model cells that replicated the conditions in diabetic hepatocytes by using the cell resealing technique, which enables the exchange of cytosol. The plasma membrane of HeLa cells was permeabilized with the streptococcal toxin streptolysin O, and cytosol that had been prepared from wild-type or db/db diabetic mice was introduced into the resulting semi-intact cells. By resealing the plasma membrane by exposure to Ca^2+^, we created WT or Db model cells, in which the cytosolic conditions replicated those of healthy or diabetic liver. Interestingly, phosphorylation of p38 MAPK was promoted, whereas the level of endosomal phosphatidylinositol-3-phosphate was decreased, in Db cells. We investigated several endocytic pathways in WT and Db cells, and found that retrograde endosome-to-Golgi transport was delayed in a p38 MAPK-dependent manner in Db cells. Furthermore, the degradation pathway of the EGF receptor from endosomes to lysosomes was enhanced in Db cells, and this did not depend on the activation of p38 MAPK. The disease model cell system should become a powerful tool for the detection of aberrant processes in cells under pathogenic conditions and for therapeutic applications.

## Introduction

Cell-based assays are increasing in importance in relation to the screening of compounds for drug discovery and investigation of their mechanisms of action. Most current cell-based assays use so-called “normal” cells, which do not reflect intracellular disease conditions. Thus, it would be useful to establish cells that model the pathogenic conditions of disease to screen potential drugs and elucidate the molecular mechanisms by which the state of diseased cells can be improved.

One possible approach is to use primary cultured cells that have been prepared from animal models of the disease of interest. However, primary cells are usually difficult to culture and cannot be used for cell-based assays due to their lack of uniformity. Another approach is to use differentiated cell lines derived from induced pluripotent stem (iPS) cells that have been obtained from patients [1], [2]. This system appears promising for the future, but it will take a long time to establish the relevant cell lines and the procedure will be difficult in practice. In addition, this system will be useful for the study of genetic disorders, but not for chronic diseases such as lifestyle-related diseases.

In the study described herein, we propose to address the limitations of current cell-based assays by using a semi-intact cell system and the cell resealing technique. Semi-intact cells are cells in which the plasma membrane has been permeabilized with a detergent or toxin [3]. For permeabilization, we use the pore-forming streptococcal toxin, streptolysin O (SLO). At 4°C, SLO binds to cholesterol in the plasma membrane. At higher temperatures, SLO oligomerizes homotypically to form pores in the plasma membrane that are 30 nm in diameter [4], [5]. The temperature-dependent pore-forming activity of SLO enables the plasma membrane to be permeabilized selectively with minimum damage to the membranes of intracellular organelles. The permeabilized cell system can be used as a type of cellular test tube, in which it is possible to conduct biochemical manipulations while maintaining the intracellular topology of organelles and the cytoskeleton. By exchanging cytoplasmic proteins with exogenously added proteins, antibodies, or cytosol that has been prepared from cells at distinct stages of the cell cycle or differentiation or in different disease states, we can modulate the intracellular environment and reconstitute various physiological phenomena in semi-intact cells. For example, by adding “pathogenic cytosol” that has been prepared from tissues of mouse models of specific diseases, together with ATP as an energy source, various biological events can be reconstructed in semi-intact cells under “pathogenic cellular conditions”. We have coupled the semi-intact cell method with green fluorescent protein (GFP)-visualization techniques, which enables us to dissect complex reaction processes in cells on the basis of cell morphology and to investigate the biochemical requirements and kinetics/dynamics of each process, for example vesicular transport under pathogenic conditions. In particular, the maintenance of the integrity of the organelles and their configuration in semi-intact cells enables us to analyze membrane trafficking or signal transduction between organelles in as intrinsic an environment as possible. To date, this system has been used to reconstitute a variety of vesicular transport pathways [6]–[11], and to investigate organelle dynamics [12]–[16], etc.

Given that the plasma membrane is permeable in semi-intact cells, this system is not optimal for analyzing signal transduction across the plasma membrane, e.g. EGF-stimulated signal transduction from the cell surface to the nucleus via the cytoplasm. In addition, the partial disruption of plasma membrane integrity makes semi-intact cells unsuitable for reconstituting endocytic processes such as the internalization of receptor/ligand complexes and the recycling and degradation of receptors and/or ligands in the lysosome. However, recent advances in cell resealing techniques have solved some of the problems that are caused by SLO-mediated pores. Walev et al. [17] found that SLO-mediated permeabilization is reversible in a manner that depends on calcium ions and microtubules. Interestingly, the resealed cells have the ability to proliferate for several days [17]. Hence, the cell resealing technique is a unique method that enables molecules delivered into living cells to exert their effects on the cells for a substantial period of time. In fact, the Collas group have used this technique successfully to perform epigenetic reprogramming of DNA methylation and histone modification within the regulatory region of Oct4 and Nanog by introducing embryonal carcinoma extracts into 293 T cells [18], [19], and have also induced 293 T cells to express T cell-like functions by exposing the cells to extracts from T cells [20]. A separate group have generated iPS cells by delivering ES cell extracts to fibroblasts [21].

The aims of the present study were: (1) to develop model cells for a given disease by incubating semi-intact cells with pathogenic cytosol prepared from the liver of model mice and resealing the cells, and (2) to analyze and identify the perturbations in biological processes, e.g. endocytic pathways, that occur at the cellular level under the pathogenic conditions. For this purpose, we introduced cytosol that had been prepared from the liver of diabetic (db/db) or wild-type (WT) mice into semi-intact HeLa cells, and then resealed them to establish Db or WT cells. The results of the morphological and biochemical analysis of the WT and Db cells indicated that p38 MAPK was activated in Db cells, which resulted in a decrease in phosphatidylinositol-3-phosphate (PI3P) in endosomes. This finding was consistent with our previous report that hydrogen peroxide (H_2_O_2_) treatment of HeLa cells decreased intracellular PI3P in a p38 MAPK-dependent manner [22]. We could reconstitute successfully the typical endocytic pathways in WT and Db cells, and identified several perturbations of endocytic pathways that occurred specifically under diabetic conditions. The disease model cell system should be beneficial for the detection of aberrant processes in cells under pathogenic conditions and for therapeutic application.

## Results

### Protocol for Preparing Good-quality Semi-intact Cells

**Figure 1:**
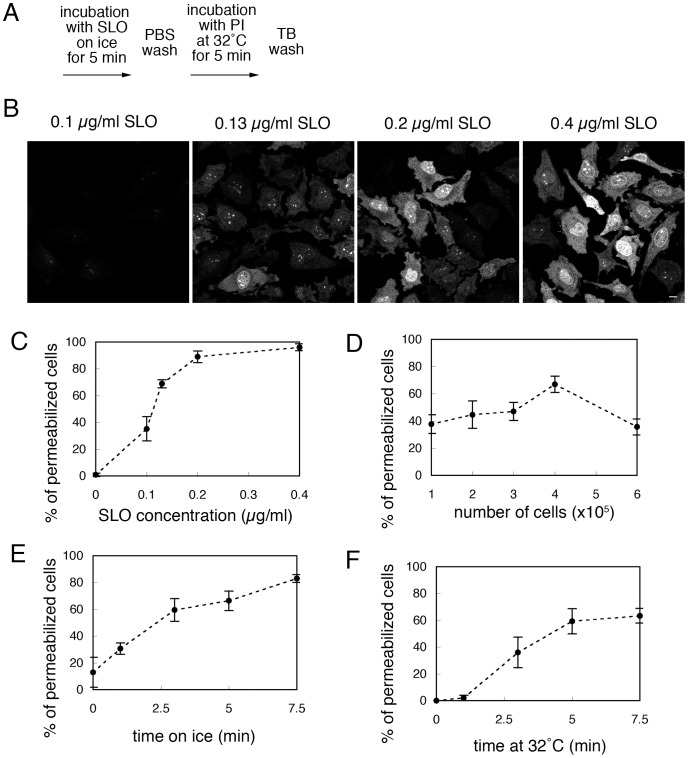
Permeabilization of HeLa cells with streptolysin O (SLO). A. Basic protocol for permeabilization of HeLa cells with SLO. B. HeLa cells were incubated with 0.10, 0.13, 0.20, or 0.40 µg/ml SLO on ice for 5 min. The cells were further incubated with transport buffer (TB) that contained propidium iodide (PI) at 32°C for 5 min, and were observed by confocal microscopy. Bar  =  10 µm. C. Dependency of permeabilization on SLO concentration. HeLa cells were treated as in B, and the means and standard deviations for the percentage of PI-positive permeabilized cells are shown in the graph. D. Dependency of permeabilization on number of cells. HeLa cells were grown on 3-cm dishes at a density of 1, 2, 3, 4, or 5×10^5^ cells per dish, and were permeabilized as in A. Means and standard deviations for the percentage of PI-positive permeabilized cells are shown in the graph. E. Dependency of permeabilization on incubation time on ice. HeLa cells were incubated with 0.13 µg/ml SLO on ice for 0.0, 1.0, 3.0, 5.0, or 7.5 min, and then further incubated with TB that contained PI at 32°C for 5 min. Means and standard deviations for the percentage of PI-positive permeabilized cells are shown in the graph. F. Dependency of permeabilization on incubation time at 32°C. HeLa cells were incubated with 0.13 µg/ml SLO on ice for 5 min, and then further incubated with TB that contained PI at 32°C for 0.0, 1.0, 3.0, 5.0, or 7.5 min. Means and standard deviations for the percentage of PI-positive permeabilized cells are shown in the graph.

The basic protocol for preparing semi-intact HeLa cells is shown in Fig. 1A. As expected, good-quality and uniformly resealed cells were obtained when the cells were permeabilized with as low a concentration of SLO as possible, because the excess SLO that remained in the culture dish after washing with TB impaired the integrity of the cell membranes. Thus, first, we reexamined the concentration of SLO that was necessary for permeabilization. HeLa cells were incubated with 0.10, 0.13, 0.20, and 0.40 µg/ml SLO on ice for 5 min, and then treated subsequently as described in the basic protocol. As shown in Fig. 1B, PI staining was found in the nucleus and throughout the cytoplasm, where DNA and RNA were located. The PI staining became stronger as the concentration of SLO was increased. We counted the number of permeabilized cells, and the means and standard deviations for the percentages of permeabilized cells at each concentration are shown in Fig. 1C. We found that > 0.2 µg/ml SLO was sufficient to permeabilize ∼90% of HeLa cells. Previously, we showed that the concentration of SLO required to give this level of permeabilization depended on the cell type [8], [13]–[16], [22], probably due to the differences in cholesterol content in the plasma membranes of different cells. Subsequent experiments were performed with 0.13 µg/ml SLO. We also evaluated the dependency of permeabilization on number of cells, and found that the permeabilization efficiency was decreased at low confluence because of the detachment of damaged semi-intact cells from the dish and also at high confluence probably due to the lack of SLO enough for permeabilization (Fig. 1D). Next, we examined the efficiency of SLO-mediated permeabilization at 0.13 µg/ml SLO by changing the time for which the cells were incubated with SLO on ice or with TB that contained PI at 32°C. Permeabilization efficiency was increased as incubation with SLO on ice proceeded (Fig. 1E). And incubation of the cells with TB that contained PI at 32°C for 5 min was sufficient for permeabilization (Fig. 1F).

### Protocol for Preparing Good-quality Resealed Cells

**Figure 2:**
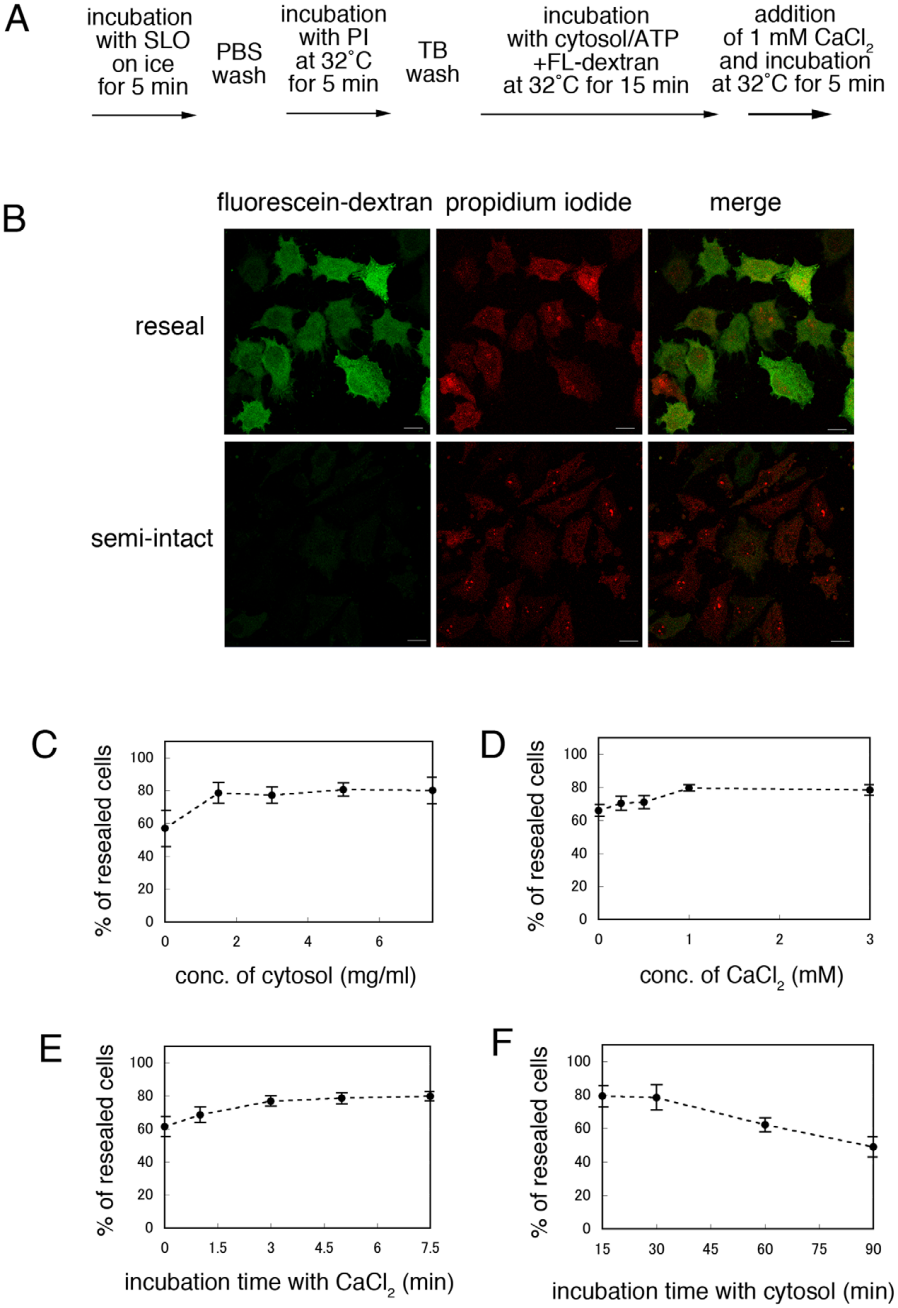
Resealing of semi-intact HeLa cells by the addition of CaCl_2_. A. Basic protocol for the resealing of semi-intact HeLa cells with CaCl_2_. B. HeLa cells were permeabilized with SLO, and were incubated with 1.5 mg/ml L5178Y cytosol, an ATP regenerating system, GTP, glucose, and fluorescein-conjugated dextran (ATP/GTP/glucose/fluorescein-dextran) at 32°C for 15 min. The cells were incubated for a further 5 min at 32°C with or without 1 mM CaCl_2_. Resealed (CaCl_2_-treated) or semi-intact (CaCl_2_-untreated) cells were incubated with DMEM supplemented with 10% FCS or TB at 37°C for 30 min. The cells were observed under a confocal microscope. Staining with both fluorescein-dextran (green) and PI (red) indicated that the cells had been resealed efficiently. Semi-intact cells were positive for PI staining but not for fluorescein-dextran. Bar  =  20 µm. C. Semi-intact HeLa cells were incubated with 0.0, 1.5, 3.0, 5.0, or 7.0 mg/ml L5178Y cytosol and ATP/GTP/glucose/fluorescein-dextran at 32°C for 15 min, and then were resealed as described in A. Means and standard deviations for the percentage of resealed cells, which were stained with both PI and fluorescein-dextran, are shown in the graph. D. Semi-intact HeLa cells were incubated with L5178Y cytosol as described in A, and then were resealed by treatment with 0.0, 0.1, 0.5, 1.0, or 3.0 mM CaCl_2_ at 32°C for 5 min. Means and standard deviations for the percentage of PI- and fluorescein-dextran-positive resealed cells are shown in the graph. E. Semi-intact HeLa cells were incubated with L5178Y cytosol as described in A, and then were resealed by treatment with 1 mM CaCl_2_ at 32°C for 0.0, 1.5, 3.0, 5.0, or 7.5 min. Means and standard deviations for the percentage of PI- and fluorescein-dextran-positive resealed cells are shown in the graph. F. Semi-intact HeLa cells were incubated with 1.5 mg/ml L5178Y cytosol and ATP/GTP/glucose/fluorescein-dextran at 32°C for 15, 30, 60, or 90 min, and then were resealed as described in A. Means and standard deviations for the percentage of PI- and fluorescein-dextran-positive resealed cells are shown in the graph.

SLO-mediated pores can be resealed rapidly in the presence of Ca^2+^ [23]. We adopted the previously described method as a basic protocol for resealing (Fig. 2A). Semi-intact HeLa cells were prepared as described in Fig. 1A. They were then incubated with cytosol prepared from murine lymphoma L5178Y cells, an ATP regenerating system, 1 mM GTP, 1 mM glucose, and 100 µg/ml fluorescein-labeled dextran at 32°C for 15 min. CaCl_2_ was added to a final concentration of 1 mM, and the cells were incubated at 32°C for a further 5 min. After incubation with medium at 37°C in 5% CO_2_ for 30 min, the resealed cells were observed by confocal microscopy (Fig. 2B). Fluorescein-dextran was used to indicate cells that were resealed without leakage (Fig.2B, fluorescein-dextran). Thus, cells that contained only PI were permeabilized but not resealed, whereas cells that contained both PI and fluorescein-dextran had undergone both permeabilization and subsequent resealing successfully. We counted the number of cells with PI staining alone [N_P_] and the number of cells with both PI and fluorescein-dextran [N_P+D_]. The percentage of [N_P+D_]/ ([N_P_] +[N_P+D_]) is shown as resealing efficiency in the graphs.

First, we examined the effect of the protein concentration of the added cytosol on the resealing efficiency. Semi-intact HeLa cells were incubated with 0.0, 1.5, 3.0, 4.5, or 7.0 mg/ml L5178Y cytosol in the presence of an ATP regenerating system, GTP, glucose, and fluorescein-dextran at 32°C for 15 min, and were resealed by the addition of 1 mM CaCl_2_ for 5 min. As shown in Fig. 2C, in the absence of cytosol, resealing occurred in ∼60% of cells. Given that measurement of the release of cytosolic LDH, showed that >80% of the cytosolic factors flowed out from the cells upon permeabilization, the remaining 20% of cytosol might be sufficient to induce resealing to a certain extent (data not shown). Cytosol at a protein concentration of more than 1.5 mg/ml was found to support effective resealing. Thus, the subsequent resealing experiments shown in Fig. 2 were performed in the presence of cytosol at a concentration of 1.5 mg /ml.

Both cytosol and Ca^2+^ had been found to be necessary for efficient resealing. However, high concentrations of Ca^2+^ would induce a variety of structural or functional perturbations in the organelles, cytoskeleton or signal transduction. Consequently, next we examined the effect of CaCl_2_ concentration on resealing efficiency. After incubating semi-intact HeLa cells in the presence of cytosol (protein concentration: 1.5 mg/ml), we added 0.0, 0.1, 0.5, 1.0, or 3.0 mM CaCl_2_ to the cells at 32°C for 5 min. We observed that cells that were resealed with >1.0 mM CaCl_2_ showed the high resealing efficiency (Fig. 2D). Resealing was not induced efficiently in the absence of CaCl_2_ or at lower concentrations of CaCl_2_ (0.1 mM). Therefore, subsequent resealing experiments were performed with 1.0 mM CaCl_2_. We also confirmed that >3 min incubation with CaCl_2_ was enough for resealing (Fig. 2E).

We also investigated the dependence of resealing on time after permeabilization. Semi-intact HeLa cells were incubated with cytosol, ATP/GTP/glucose, and fluorescein-dextran at 32°C for 15, 30, 60, or 90 min. The cells were then resealed and observed by confocal microscopy. Incubation with cytosol for 15 or 30 min did not cause a major effect on cell morphology (data not shown). However, the percentage of resealed cells was decreased when the cells were incubated with cytosol for 60 or 90 min (Fig. 2F), and we sometimes observed the disruption of resealed cells (data not shown). These results suggested that a longer period of incubation with cytosol reduced the resealing efficiency, probably due to damage or disruption to cellular components. We also tested the requirements for an ATP regenerating system on the resealing process. Semi-intact HeLa cells were incubated with L5178Y cytosol and fluorescein-dextran in the presence or absence of ATP/GTP/glucose at 32°C for 15 min, and were resealed. Interestingly, ∼75 % of resealed cells without ATP/GTP/glucose underwent cell death, even in the presence of cytosol, which indicated that these molecules were required for cell viability (data not shown).

**Figure 3:**
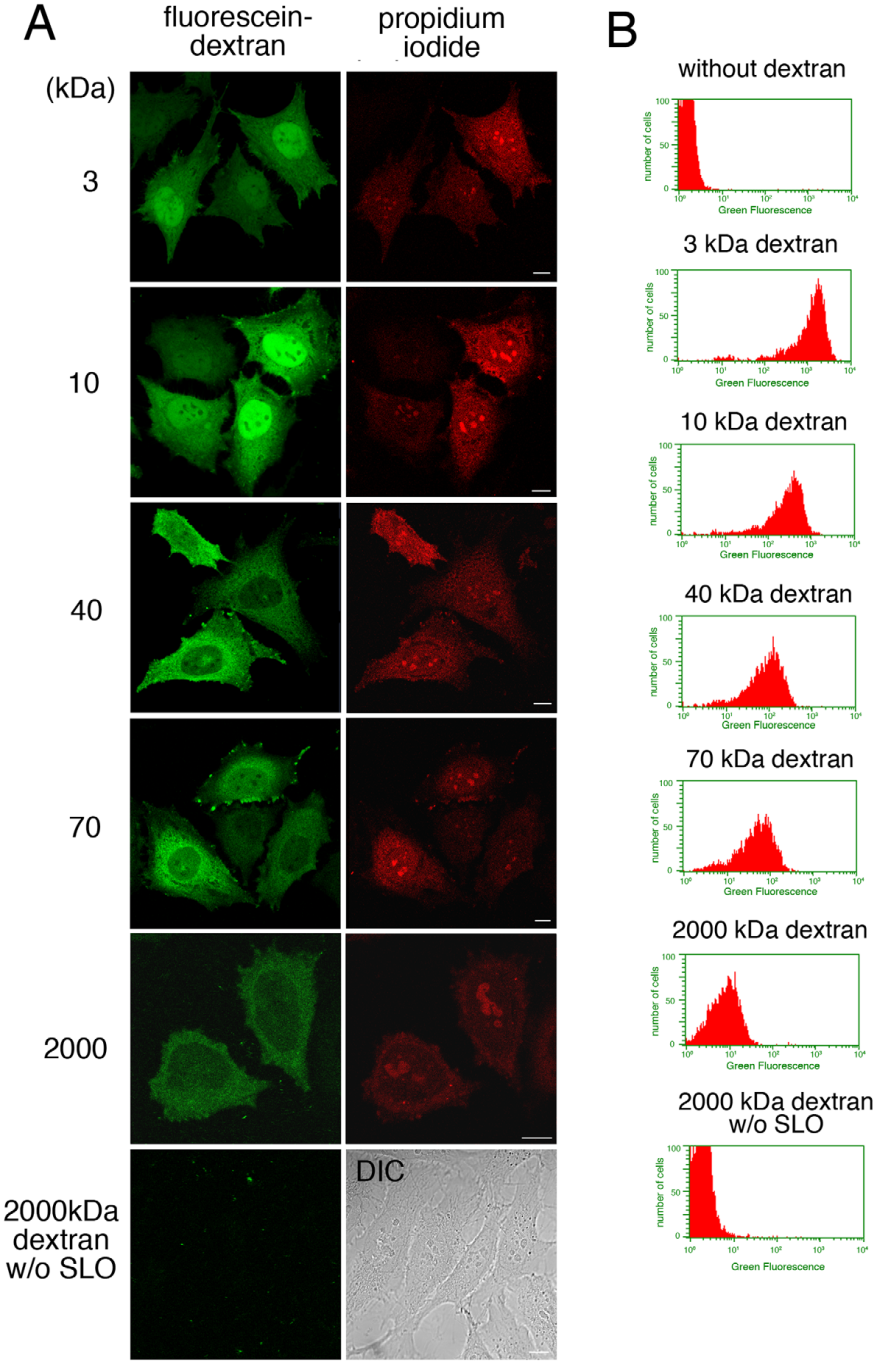
Introduction of fluorescein-dextran of different molecular weights into resealed cells. A. HeLa cells were incubated with or without (2000 kDa dextran w/o SLO) 0.13 µg/ml SLO on ice for 5 min. After wash with PBS three times, the cells were further with transport buffer containing propidium iodide at 32°C for 5 min. Semi-intact HeLa cells were incubated with 1.5 mg/ml L5178Y cytosol, an ATP regenerating system, GTP, glucose, and 100 µg/ml fluorescein-dextran of 3, 10, 40, 70, or 2000 kDa at 32°C for 15 min, and then were resealed by treatment with 1 mM CaCl_2_ at 32°C for 5 min. After incubation with DMEM supplemented with FCS for 30 min, the cells were observed by confocal microscopy. Since the cells without SLO treatment did not contain the fluorescence of propidium iodide, differential interference contrast (DIC) image was shown. Bar  =  10 µm. B. HeLa cells were treated as described in A, were trypsinized, and were subjected to flowcytometry. The histograms of fluorescein fluorescence of dextran with different molecular weight in PI-positive cells were shown.

In addition to the resealing efficiency, information about the size of the molecules that could be introduced into and retained in the resealed cells could be useful. To test this, semi-intact HeLa cells were incubated with 3, 10, 40, 70, or 2000 kDa dextran conjugated with fluorescein in the presence of cytosol and ATP/GTP/glucose at 32°C for 15 min. The cells were then resealed and observed by confocal microscopy. To our surprise, as shown in Fig. 3A, all the molecular sizes of fluorescein-dextran that we tested could be introduced into the semi-intact cells and retained in the resealed cells. Dextran of ≥40 kDa was observed throughout the cytoplasm, whereas dextran of 3 or 10 kDa diffused into the nucleus. This result was consistent with the reports of others [24], [25], and suggested that the nuclear barrier was maintained in the resealed cells. To demonstrate the retention of dextran in resealed cells more clearly, we performed flow cytometry using the Guava easyCyte 8 HT flow cytometry system. Fig. 3B shows the histogram for fluorescein fluorescence of PI-positive cells. As shown in Fig. 3B, the fluorescence intensity of dextran-containing cells increased as the molecular weight of the introduced dextran decreased (i.e. as the number of molecules in the reaction mixture increased, given that a fixed concentration of dextran was used). The average fluorescein fluorescence intensity of the population of resealed cells that contained 2000 kDa dextran was 10.93, whereas the intensity was 3.33 in resealed cells that did not contain fluorescein-dextran, and was 3.32 in non-permeabilized cells that were incubated with fluorescein-dextran (Fig. 3B). Furthermore, we did not detect non-specific binding of 2000 kDa dextran to plasma membrane or encodytosis of dextran in non-permeabilized cells (Fig. 3A, 2000 kDa dextran w/o SLO). We also confirmed that the autofluorescence of the cells did not interfere the observation of fluorescein-dextran under microscopy. These results suggested that, although the fluorescence signal was weak, 2000 kDa dextran was indeed retained in resealed cells.

We observed that the fluorescence intensity of PI and dextran varied from cell to cell, and flow cytometric analysis showed some deviation in the fluorescence intensity of PI and dextran (Fig. 3B). However, we did not detect a significant correlation between the fluorescence intensity of PI and that of dextran (R^2^ value was 0.3478). This result suggested that the efficiency of permeabilization and/or resealing differed to some extent among resealed cells, but did not indicate that the greater the intensity of PI fluorescence, the more dextran entered into cells or the more efficiently cells were resealed.

We also checked the structures of organelles and the cytoskeleton in resealed cells. Resealed cells were prepared according to the protocol in Fig. 2A, and were incubated with medium at 37°C for 30 min. Intact or resealed HeLa cells were stained with antibodies against GM130 (cis-Golgi marker), ERGIC53 (ER-Golgi intermediate compartment marker), Lamp1 (lysosome marker), cytochrome C (mitochondrial marker), EEA1 (early endosome marker), actin (actin filament marker), tubulin (microtubule marker), or Hoechst 33 (nuclear marker), and ER tracker (ER marker) (Fig. S1). As we added the fluorescently labeled dextran with cytosol and ATP/GTP/glucose, we were able to distinguish resealed cells, which were fluorescently labeled with dextran, from non-resealed cells easily under a fluorescence microscope. We found that the morphology of most of the organelles remained intact in the resealed cells. However, we observed that mitochondria were accumulated in the perinuclear region in the resealed cells (Fig. S1, cytochrome C). In addition, arrays of microtubules and actin filaments were found to be reduced slightly (Fig. S1, actin and tubulin), but they were restored with increasing incubation time in medium (data not shown).

### Healthy and Pathogenic Model Cells: Changing Intracellular Conditions to the Diabetic Liver State by the Cell Resealing Technique

In the present study, we established resealed cells in which the intracellular condition mimicked that of liver cells in mice with diabetes. The protocol was as follows. First, we prepared cytosol from the liver of wild-type mice, referred to as WT cytosol, and from the liver of db/db mice, referred to as Db cytosol. The db/db mouse is homozygous for a point mutation in the gene for the *leptin* receptor, and is used as a model of obesity and diabetes. Semi-intact HeLa cells were incubated with WT or Db cytosol (3 mg/ml) in the presence of ATP/GTP/glucose and fluoresein-dextran at 32°C for 30 min, and then were resealed. The cells were incubated further with medium at 37°C in 5% CO_2_ for 30 min to restore cell viability. The resealed cells that contained WT cytosol or Db cytosol were called WT model cells or Db model cells, respectively.

**Figure 4:**
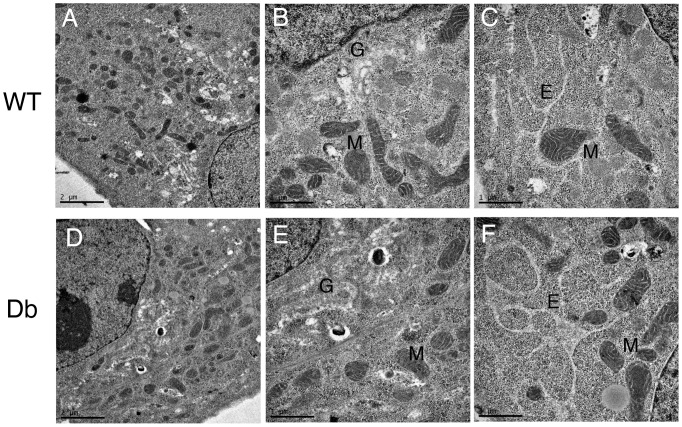
Electron microscopic observation of WT and Db cells. Resealed WT (A, B, and C) and Db (D, E, and F) cells were observed by electron microscopy. G, M, or E indicates the Golgi apparatus, mitochondria, or endoplasmic reticulum, respectively. No significant difference in organelles morphology was observed between WT and Db cells. Bar  =  2 µm (A and D) or 1 µm (B, C, E, and F).

First, we examined the morphological integrity of organelles and the cytoskeleton in WT and Db model cells by immunofluorescence. As shown in Fig. S2, almost all organelles and the cytoskeleton in Db cells were morphologically indistinguishable from those in WT cells. Interestingly, staining of EEA1, a marker of early endosomes, was slightly fainter in Db cells than in WT cells, although the morphology of the endosomes did not change (Fig. S2, EEA1). To further examine the intracellular structures at the ultrastructural level, we performed electron microscopic observation of WT and Db cells. Although the Golgi apparatus was swollen slightly in both WT and Db cells compared to that in intact cells, no significant differences in organelle morphologies were detected between WT and Db cells by electron microscopic observation (Fig. 4).

**Figure 5:**
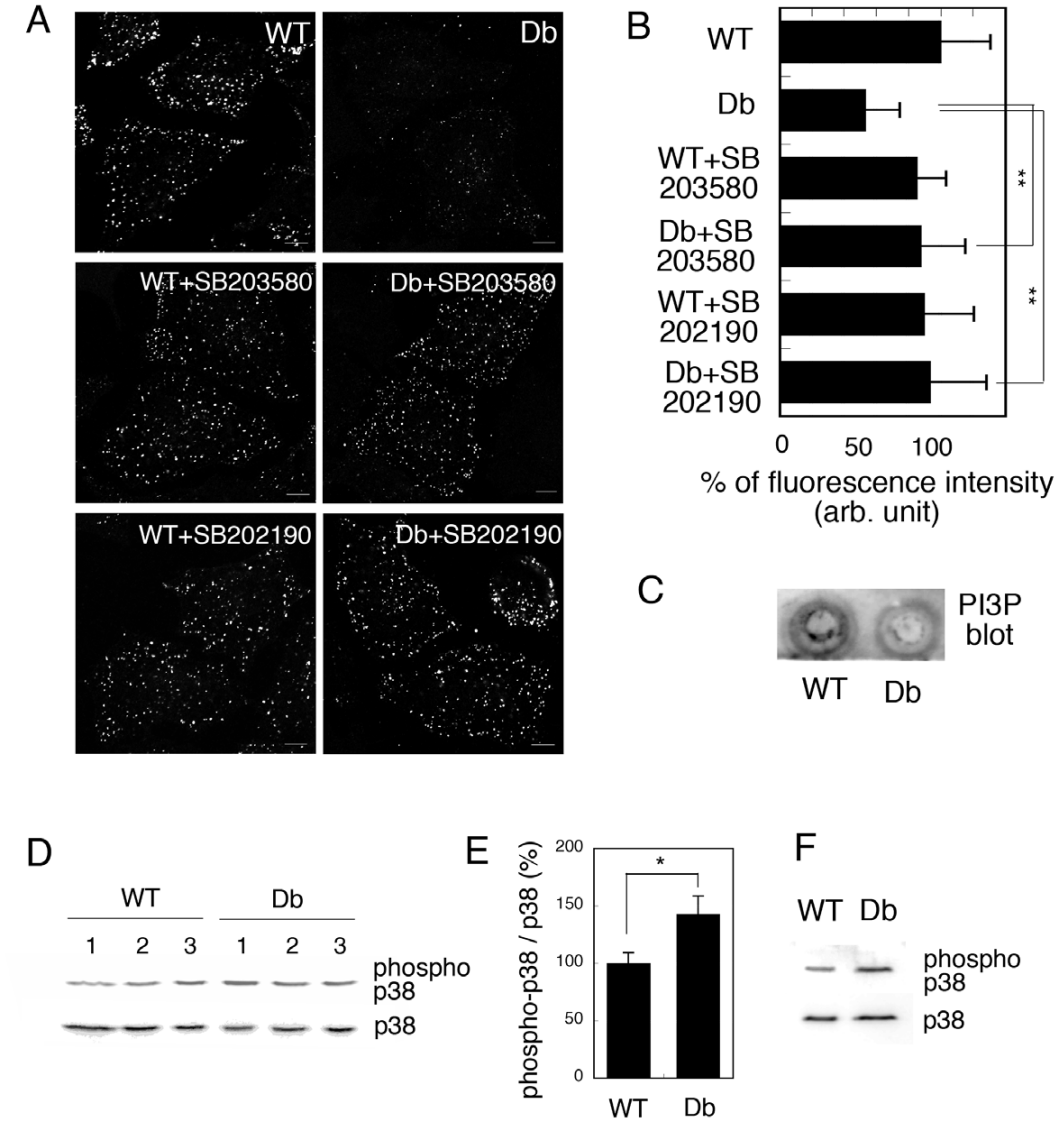
Intracellular PI3P was decreased in a p38 MAPK-dependent manner in Db cells. A. HeLa cells were pretreated with or without 2 µM SB203580 or 2 µM SB202190 at 37°C for 60 min. Semi-intact HeLa cells were incubated with 3 mg/ml WT or Db liver cytosol, an ATP regenerating system, GTP, and glucose in the presence or absence of 2 µM SB203580 or SB202190 at 32°C for 30 min, and then for a further 15 min at 32°C after the addition of 1 µg of GST-2xFYVE recombinant protein. The cells were fixed and GST-2xFYVE was visualized with Alexa488-conjugated antibodies against GST. Bar  =  10 µm. B. The fluorescence intensity of GST-2xFYVE was measured as described in Materials and Methods, and the means and standard deviations for the fluorescence intensity are shown in the graph. We performed three independent experiments and counted 100 cells in each experiment. Data were analyzed using one-way ANOVA and Dunnett’s post hoc test, and the *P* value was < 0.01 (**). C. Measurement of PI3P content in WT and Db cells by lipid blot. D. Western blotting of p38 MAPK and phosphorylated p38 MAPK in liver lysates from three WT or three Db mice. E. The intensities of the bands shown in C were measured and the relative proportion of phosphorylated p38 is shown as a percentage. Means and standard deviations for the relative proportion are shown in the graph. F. Western blotting of p38 MAPK and phosphorylated p38 MAPK in WT and Db cells.

EEA1 is known to be targeted to early endosomes by binding to phosphatidylinositol-3-phosphate (PI3P) through its FYVE domain [26], [27]. Therefore, we examined the level of intracellular PI3P in WT and Db cells by the FYVE-targeting assay. We established this assay previously to measure semi-quantitatively by immunofluorescence the amount of intracellular PI3P using probes that contain the PI3P binding (FYVE) domain [22]. Semi-intact HeLa cells were incubated with WT or Db liver cytosol for 30 min, then further with GST-2xFYVE proteins for 15 min to label the intracellular PI3P. The cells were fixed and were immunostained with anti-GST antibody. As shown in Fig. 5A and 5B (WT and Db), GST-2xFYVE-positive punctate endosome structures were clearly visible in WT model cells, but were only faintly detectable in Db model cells. To complement the result of FYVE-targeting assay biochemically, we performed lipid blot analysis of PI3P in WT and Db cells (Fig. 5C). The lipid from WT and Db cells was extracted and spotted onto the membrane. Then, PI3P was detected by using GST-2xFYVE proteins (Fig. 5C). We found that the PI3P content in Db cells was decreased to 56.59 ± 5.38% of that in WT cells. We verified the data by applying *t*-test, and the *P* value was <0.05. These results indicated that PI3P content was decreased in Db cells.

We further investigated the factor(s) that induced the Db cytosol-dependent decrease in the amount of PI3P at endosomes and focused on the correlation between p38 MAPK activity and endosomal PI3P. Previously, we had found that the amount of endosomal PI3P was decreased by H_2_O_2_-induced activation of p38 MAPK in HeLa cells [22]. To test this, we pretreated HeLa cells with 2 µM SB203580, an inhibitor of p38 MAPK, permeabilized them, and then incubated them with WT or Db cytosol in the presence of SB203580. We used SB203580 at the concentration of 2 µM since SB203580 at 2 µM is suitable to inhibit p38 MAPK activity without activating other kinases according to the paper by Lali et al. [28]. We then subjected the cells to the FYVE-targeting assay. Interestingly, SB treatment restored the targeting of GST-2xFYVE in Db cells (Fig. 5A and 5B, Db+SB203580). The similar result was obtained by using another p38 MAPK inhibitor, SB202190, at the concentration of 2 µM (Fig. 5A and 5B, Db+SB202190). These results suggested that the activation of p38 MAPK in Db cytosol played a crucial role in the decrease in the amount of PI3P in Db cells, which resulted in the inhibition of FYVE targeting to endosomes.

These results further suggest that p38 MAPK should be activated in diabetic liver. Consequently, we prepared liver lysates from three WT and three db/db mice, and examined the activation of p38 MAPK in each lysate by western blotting using antibodies against p38 or phosphorylated (activated) p38 MAPK. As shown in Fig. 5D, the phosphorylation of p38 MAPK was increased in Db liver lysates compared to that in WT lysates. We quantified the ratio of phosphorylated (activated) p38 MAPK to total expressed p38 MAPK in each sample and found that the proportion of phosphorylated p38 MAPK was ∼1.5 fold greater in the Db liver lysate than in the WT lysate (Fig. 5E). This is consistent with several reports that showed that phosphorylation of p38 MAPK was increased in db/db and ob/ob mice, as well as mice in which obesity had been induced by a high-fat diet [29], [30], [31]. We also examined the phosphorylation of p38 MAPK in resealed WT and Db model cells by western blotting, and confirmed that p38 MAPK was more phosphorylated in Db cells than in WT cells (Fig. 5F). These results suggested that p38 MAPK was activated in the diabetic liver condition, which would cause the decrease in endosomal PI3P in Db cells.

### Recycling of Transferrin in Db Cells was Indistinguishable from that in WT Cells

As mentioned previously, H_2_O_2_ treatment decreases the level of intracellular PI3P in a p38 MAPK-dependent manner, and inhibits a broad range of endocytic pathways [22]. Having found that a similar perturbation of the amount of PI3P and precise recruitment of the FYVE domain-containing protein EEA1 to early endosomes occurred in Db cells, we next examined the effect of Db cytosol on a variety of endocytic pathways. First, we examined the recycling pathway of transferrin (Tf) (Fig. S3). As shown in Fig. S3A, Tf could bind to the plasma membrane (0 min), become internalized (10 min), and be recycled back to the plasma membrane (20 min) in both WT and Db cells with similar kinetics. It should be noted that reconstitution of the endocytosis and recycling of Tf is difficult in semi-intact cells owing to the disrupted plasma membrane, but resealing of the plasma membrane enabled us to establish suitable assays in WT and Db cells. We measured the fluorescence intensity of Alexa488-Tf in each sample of WT and Db cells at 0 min. As shown in Fig. S3B, the amount of bound Tf in WT cells was almost identical to that in Db cells, which suggested that Db cytosol had no effect on the number of Tf receptors and their affinity for Tf. We also observed that the kinetics of internalization followed by recycling of Tf in Db cells was indistinguishable from that in WT cells (Fig. S3C).

To obtain more quantitative data on Tf recycling, we performed a flow cytometry assay. By measuring the fluorescence of dextran, we confirmed that 89.8 ± 2.7 % of the cells were resealed under the experimental conditions. As shown in Fig. S3D and S3E, the amount of Tf that bound to the plasma membrane was almost identical in WT and Db cells (0 min). After 10 and 20 min, Alexa488-Tf had decreased to ∼55% and ∼12%, respectively, in both WT and Db cells (Fig. S3E), which suggested that internalization and recycling occurred at a similar rate in the two cell types.

### Transport of Cholera Toxin from Endosomes to the Golgi was Perturbed in Db Cells in a p38 MAPK-dependent Manner

**Figure 6:**
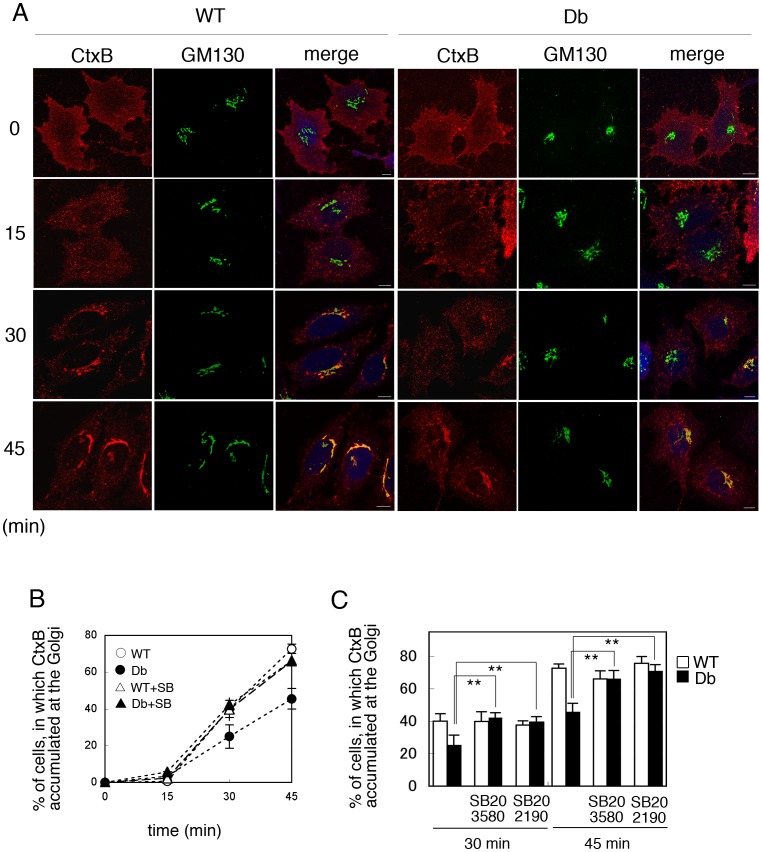
Retrograde transport of Cholera toxin B subunit (CtxB) in WT and Db cells. A. Semi-intact HeLa cells were incubated with 3 mg/ml WT or Db liver cytosol in the presence of ATP/GTP/glucose and Alexa647-conjugated dextran (10 kDa, blue) at 32°C for 30 min, and then resealed by addition of 1 mM CaCl_2_ for 5 min. After incubation with DMEM supplemented with 10% FCS for 30 min, the cells were treated with 2 µg/ml Alexa546-conjugated CtxB (red) on ice for 30 min, and then incubated with medium at 37°C for 0, 15, 30, and 45 min. The cells were fixed, were immunostained with antibodies against GM130 (green), and were observed by confocal microscope. Bar  =  10 µm. B. We counted the number of cells in which CtxB was accumulated at the Golgi on the basis of the colocalization of CtxB with GM130, after a 0, 15, 30, and 45 min chase in WT (○) and Db (•) cells with or without treatment of 2 µM SB203580 (△, WT+SB; ▴, Db+SB). The means and standard deviations for the percentages of these cells are shown in the graph. Three independent experiments were performed and we counted 300 cells in each experiment. C. CtxB transport assay was performed in WT or Db cells treated with or without 2 µM SB203580 or 2 µM SB202190. In 30 or 45 min after internalization of CtxB by incubating cells at 37°C, the cells were fixed and the number of cells in which CtxB was accumulated at the Golgi was counted. The means and standard deviations for the percentages of the cells are shown in the graph. Data were analyzed using one-way ANOVA and Dunnett’s post hoc test, and the *P* value was < 0.01 (**), which indicated that transport of CtxB to the Golgi was significantly delayed in Db cells compared to that in Db cells treated with SB203580 or SB202190.

Next we examined the retrograde transport of cholera toxin from the plasma membrane to the Golgi apparatus in WT and Db cells. WT and Db cells were incubated with Alexa546-conjugated cholera toxin B subunit (Alexa546-CtxB) on ice for 30 min, and then incubated further with medium at 37°C for 0, 15, 30, and 45 min. The cells were cells were fixed and immunostained with antibody against GM130, a cis-Golgi marker, and were observed by confocal microscopy. We could distinguish the resealed cells from non-resealed cells by the labeling of resealed cells with Alexa647-conjugated dextran, and compared the difference in Ctx transport between WT and Db cells. We counted the number of cells in which CtxB had colocalized with GM130, at each time point. We observed that in both WT and Db cells, Alexa546-CtxB bound to the plasma membrane, was internalized to punctate endosomal structures, and then was further transported to the Golgi apparatus in the perinuclear region, even in resealed cells (Fig. 6A). As shown in Fig. 6B, retrograde transport of CtxB to the Golgi was delayed in Db cells as compared with WT cells. We observed that the internalization of Ctx to the punctate endosomal structures was indistinguishable between WT and Db cells, suggesting that the internalization of CtxB from the plasma membrane to the endosomes occurred to the same extent in WT and Db cells. These results indicated that the transport of CtxB from the endosome to the Golgi was substantially inhibited in Db cells.

We reported previously that retrograde transport from endosomes to the Golgi is inhibited in H_2_O_2_-treated HeLa cells in a p38 MAPK-dependent manner [22]. To examine whether the activation of p38 MAPK is responsible for the delay of CtxB transport in Db cells, we tested the effect of the p38 MAPK inhibitor SB203580 on the inhibition of CtxB transport in Db cells. In this experiment, HeLa cells were treated with 2 µM SB203580 for 60 min, permeabilized, and incubated with WT or Db cytosol in the presence or absence of SB203580. After resealing, the CtxB transport assay was performed in the presence or absence of SB. As shown in Fig. 6B and 6C, SB203580 treatment restored CtxB transport in Db cells, whereas it had no effect on the transport in WT model cells (Fig. 6B and 6C, WT+SB203580, Db+SB202190). Treatment with another p38 MAPK inhibitor, SB202190, at the concentration of 2 µM also restored CtxB transport to the Golgi (Fig. 6C, Db+SB202190). These results suggested that under diabetic conditions, activated p38 MAPK inhibited the retrograde transport of CtxB from endosomes to the Golgi.

### Internalization and Degradation of the EGF Receptor was Promoted in Db Cells as Compared with WT Cells

**Figure 7:**
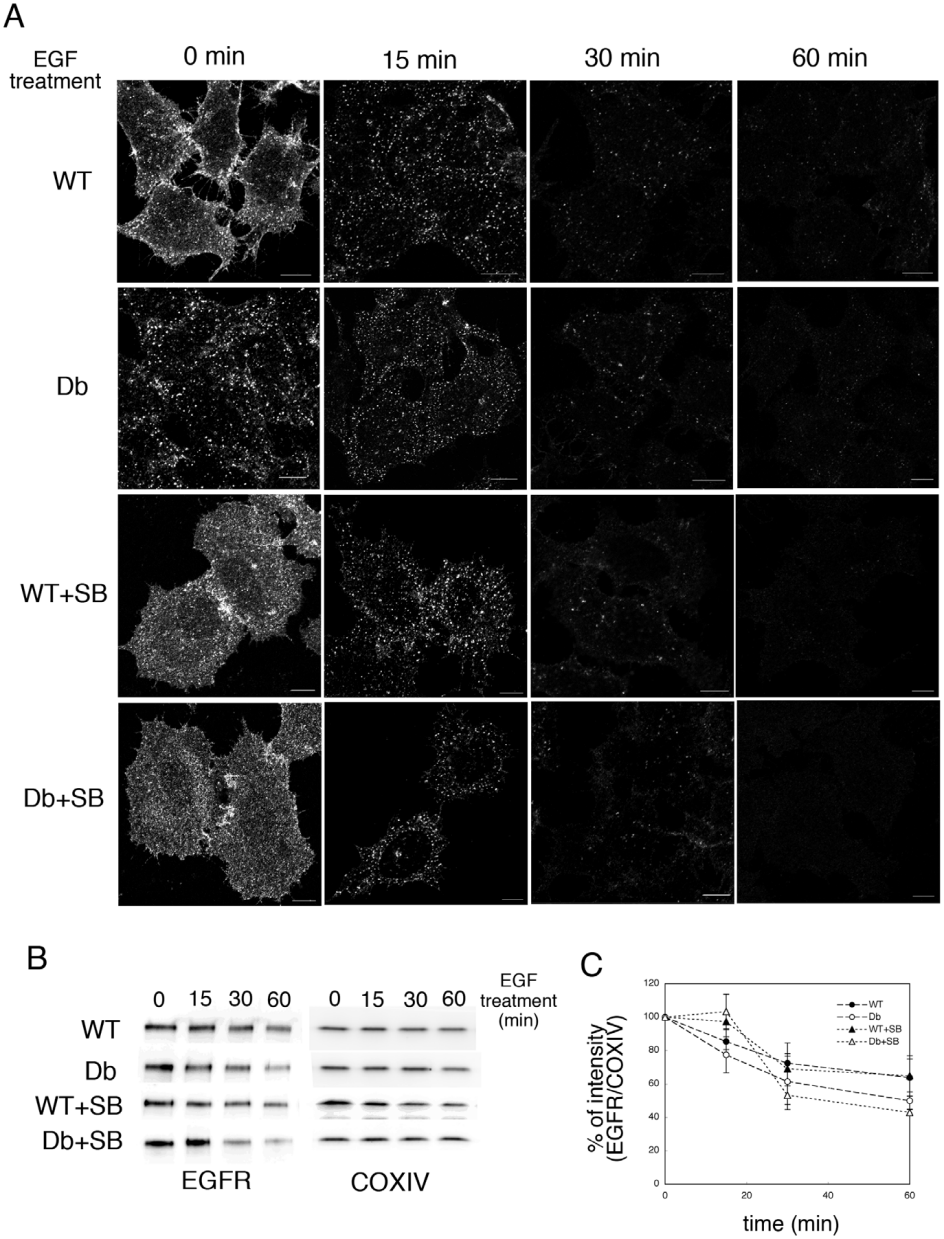
Degradation of EGFR in WT and Db cells. A. HeLa cells were preincubated with DMEM without serum overnight, and were incubated without (WT and Db) or with (WT+SB and Db+SB) 2 µM SB203580 for 60 min. Semi-intact HeLa cells were incubated with WT or Db liver cytosol that contained Alexa546-conjugated dextran, and were resealed. After incubation with DMEM in the presence or absence of SB203580 for 30 min, the cells were treated with 10 ng/ml EGF, and then incubated with medium at 37°C for 0, 15, 30, and 60 min. The cells were fixed, and stained with anti-EGFR antibody. We were able to distinguish resealed cells, which were fluorescently labeled with dextran, from non-resealed cells easily under a fluorescence microscope. Bar  =  10 µm. B. HeLa cells were treated as described in A, lysed, and subjected to Western blotting using antibodies against EGFR and COX VI. C. Means and standard deviations for the band intensities of EGFR/COX VI are shown in the graph. We performed three independent experiments and verified the results by applying Student’s *t*-test. We found that the *P* value was > 0.05, which indicated that treatment with 2 µM SB203580 did not affect the enhanced degradation of EGFR by Db cytosol.

Next, we examined the endocytic transport of the complex formed by EGF and the EGFR in WT and Db cells. EGFR is down-regulated upon EGF binding by internalization and delivery to the lysosome. First, we investigated the kinetics of EGFR degradation in WT and Db cells. After incubation with FCS-free medium overnight, the cells were permeabilized and incubated with WT or Db cytosol in the presence of ATP/GTP/glucose and TMR-dextran. The cells were resealed, incubated with DMEM without FCS for 30 min, and then stimulated with 10 ng/ml EGF for 0, 15, 30, and 60 min. After incubation, the samples were subjected to immunofluorescence analysis using an anti-EGFR antibody. Interestingly, even in the 0-min sample, a substantial EGFR signal was observed at both the plasma membrane and endosomes in WT and Db cells (Fig. 7A, WT and Db, 0 min). In addition, the amount of internalization of EGFR in Db cells was greater than that in WT cells (Fig. 7A, 0 min, WT and Db). This result indicated that the internalization of EGFR in the absence of EGF seemed to be enhanced in Db cells as compared with WT cells. To examine the involvement of p38 MAPK in EGFR internalization during the resealing process (i.e., in the 0-min sample), EGFR internalization was analyzed in WT and Db cells in the presence of 2 µM SB203580. As shown in Fig. 7A (WT+SB and Db+SB), SB treatment completely inhibited the internalization of EGFR during the resealing process (without/before EGF treatment). This result indicates that even without EGF stimulation, EGFR was internalized from the plasma membrane during resealing.

Within 15 min after the addition of EGF, almost all the EGFR was internalized to endosomes, and then the amount of EGFR in endosomes decreased gradually at 30 and 60 min (Fig. 7A). Western blot analysis using an antibody against EGFR revealed that the degradation of EGFR was promoted in Db cells as compared with WT cells: 36.2 ± 11.1 % of EGFR was degraded in WT cells at 60 min after EGF stimulation, whereas 50.1 ± 5.3 % was degraded in Db cells (Fig. 7B and 7C, WT and Db). To examine the involvement of p38 MAPK in EGFR degradation, an EGFR degradation assay was performed in WT and Db cells in the presence of 2 µM SB203580. In the presence of SB, EGFR was internalized within 15 min after the addition of EGF in both WT and Db cells, and was degraded within 30 and 60 min after EGF treatment (Fig. 7A, WT+SB and Db+SB, 60 min). We measured the rate of degradation of EGFR by western blotting and found that EGFR was degraded faster in Db+SB cells than in WT+SB cells (Fig. 7B and 7C, WT+SB and Db+SB). Thus, after complete resealing followed by further incubation to restore cell viability, the resealed Db cells showed an enhanced degradation of EGFR upon EGF stimulation, but the degradation was independent of p38 MAPK. These results suggested that the internalization and degradation of EGFR upon EGF stimulation were enhanced under diabetic conditions, but this was not regulated by p38 MAPK. The Db cytosol might have the ability to promote the internalization of EGFR in a p38 MAPK-dependent manner in the absence of EGF stimulation, as well as a distinct ability to promote the internalization and degradation of EGFR in the presence of EGF stimuli.

## Discussion

### Basic Protocol for the Resealing of HeLa Cells

The resealing efficiency and the integrity of resealed cells is important for the reliability and reproducibility of cell-based assays that use such cells. In the present study, the resealing efficiency was estimated from the percentage of cells in which exogenously added fluorescein-dextran remained 30 min after resealing (Fig. 2B). We found that the semi-intact cells that were damaged severely by treatment with a high concentration of SLO showed damaged microtubules and actin filaments (data not shown). Such cells were no longer resealed effectively by CaCl_2_ treatment. The resealing process requires membrane trafficking functions, such as endocytosis [23], exocytosis [32], and ectocytosis [33], which are largely dependent on the integrity of the cytoskeleton and its associated motor proteins. Thus, the integrity of the cytoskeleton is one important factor in relation to ensuring a high resealing efficiency. To diminish the damage to the cytoskeleton by SLO treatment, we found that 0.13 mg/ml SLO was sufficient and suitable for the permeabilization of HeLa cells when other conditions (e.g. the time of incubation of cells with SLO or with TB at 37°C for resealing) were kept constant (Fig. 2).

Calcium ions are essential for cell resealing, but high concentrations of intracellular Ca^2+^ (≥10 µM in HEK293 cells) induce cell death [34]. In addition, the elevation of intracellular Ca^2+^ induces the destabilization of α-actin polymer in a myosin light chain kinase-dependent manner [35]. Hence, high levels of Ca^2+^ might inhibit the resealing of semi-intact cells. However, we found no significant changes in organelle morphology and the organization of actin filaments were observed after resealing, which suggested that the calcium treatment had little effect on the organization of cytoskeletal elements such as microtubules and actin filaments. Thus, in all subsequent experiments in the study, we used calcium at a concentration of 1 mM, which was sufficient for the resealing of semi-intact HeLa cells (Fig. 2D).

In addition to Ca^2+^, cytosol is also essential for the resealing process. We found that L5178Y cytosol at a concentration of 1.5 mg/ml was sufficient for resealing (Fig. 2C), but for reasons of convenience, we used the cytosol at a protein concentration of 3.0 mg/ml.

### Structural/morphological Integrity of the Resealed Cells

In the present study, successful resealing was confirmed by analyzing the retention of fluorescein-dextran of various molecular weights that ranged from 3 kDa to 2,000 kDa in resealed cells. Using this approach, we found that resealing was usually accomplished immediately, within at least 5 min after treatment with CaCl_2_/cytosol (Fig. 2E). For example, even 3 kDa fluorescein-dextran was trapped in the resealed cells within 5 min after resealing with CaCl_2_/cytosol, and was retained in the cells subsequently for more than 48 hrs (Fig. 3 and data not shown).

As shown in Fig. 3, even 2,000 kDa fluorescein-dextran could be indeed introduced into semi-intact HeLa cells, although the fluorescence signal of retained dextran was weak. This finding was unexpected because SLO-mediated pores are ∼30 nm in diameter [4], [5], which should allow the entry of molecules up to ∼150 kDa, such as antibodies, but exclude larger molecules. The mechanisms of how such a large molecule could pass through SLO-mediated pores have not been elucidated yet. We assume that the conformation of the molecules will affect the entry process, with string-shaped dextran polymer able to pass through the pores. Another possibility is that SLO-mediated pores fuse with one another temporarily to make larger pores. Thus, the ability to cross the SLO-mediated pores might depend on the shape or conformation of the molecules. Therefore, it would be necessary to test the entry of various kinds of probes other than dextran to examine the requisite characteristics of the molecules to enter into resealed cells through SLO-mediated pores. In addition, electron microscopic analysis would be required to elucidate the mechanisms involved.

Immunofluorescence analysis using antibodies against marker proteins for several organelles revealed that the resealing protocol had little effect on organelle morphology, using intact cells as a control (Fig. S1). One exception was mitochondria, for which both shape and localization were affected. In intact HeLa cells, mitochondria showed an elongated structure with an average length of approximately 5 µm and were localized throughout the cytoplasm (Fig. S1, cytochrome C, intact). In contrast, just after resealing, the mitochondria in resealed cells appeared to be fractionated and slightly swollen, and they were accumulated in the perinuclear region (Fig. S1, cytochrome C, resealed). The fractionated and swollen mitochondria were restored to normal after incubation with medium in a CO_2_ incubator for 45∼60 min (data not shown). Furthermore, at electron microscopic level, the Golgi apparatus was also swollen slightly (Fig. 4), indicating that the some perturbation of ion balance in semi-intact or resealed cells might cause such swelling of several organelles.

Permeabilization by SLO activated several stress-response kinases, such as p38 MAPK [36], [37], [38], JNK [37], and NFκB [39], which was followed by the release of IL-6, IL-8, and tumor necrosis factor alpha [36], [39]. In particular, Kloft et al. [38] demonstrated that the loss of intracellular potassium ions owing to the formation of pores was responsible for the activation of p38 MAPK. We also investigated the status of the signaling pathways of p38 MAPK, JNK, and p42/44 MAPK, by western blotting using phospho-specific antibodies against these kinases. As shown in Fig. S4 (p38 MAPK and p42/44 MAPK), phosphorylation of p38 MAPK and p42/44 MAPK was enhanced in resealed cells as compared with intact cells, but decreased gradually with ongoing incubation, although the phosphorylated forms were not completely eliminated. The phosphorylation status of JNK in resealed cells was indistinguishable with that in intact cells (Fig. S4, JNK), although this might be dependent on cell type. The stress response might be reduced in resealed cells because incubation with medium that contained a high concentration of potassium ions prevented p38 MAPK activation upon SLO treatment [38]; however, complete elimination of stress-induced events would be difficult. Therefore, we should consider the effect of permeabilization and resealing on the basal intracellular environment when reconstituting biological processes.

### Preparation of Disease or Healthy Model Cells

In this study, we established a basic protocol for preparing disease or healthy model cells from semi-intact HeLa cells using WT and Db cytosol. WT and Db cytosol, which were prepared from the liver of WT and db/db mice, respectively, had no effect on the resealing efficiency (79.56 ± 4.15% and 80.39 ± 6.62%, respectively, by flow cytometry).

The type of cells that are used to generate resealed cells is an important consideration for the establishment of disease model cells or cell-based assays. In the present study, we developed a resealed cell system to analyze intracellular events under diabetic conditions by adding cytosol from the liver of diabetic mice. Thus, it might be better to use hepatocytes to generate the resealed cells. With this aim, we first tried to use HepG2 cells, a commonly-used human hepatoma, for the resealed cells. Unfortunately, however, we found that the resealing of semi-intact HepG2 cells immediately caused cell death, whereas semi-intact HepG2 cells appeared to retain the intracellular structures without obvious severe damage (data not shown). We are now looking for hepatocyte cell lines that are suitable for the generation of resealed cells.

Therefore, we used HeLa cells in the study for the following reasons. 1) HeLa cells can be handled easily and we were able to use the technical expertise for the permeabilization of HeLa cells that we had acquired in our previous studies. 2) In our paper published in 2011 [22], we established the FYVE-targeting assay to measure intracellular PI3P in HeLa cells, as well as several assays for endocytic pathways (EGFR degradation, CtxB retrograde transport, and transferrin recycling). These assays could be applied to the present study. 3) We think that the model cell system established in the present study is the prototype for future studies. Thus, the results and experimental conditions for permeabilization/resealing that were established with HeLa cells should provide useful information for other researchers who adopt the resealing cell technique, because HeLa cells are commonly used by many researchers.

Interestingly, we observed that the proportion of activated (phosphorylated) p38 MAPK was increased in Db cells compared to that in WT cells (Fig. 5F). The increase in p38 MAPK phosphorylation could be attributed to both the stress response of the cells against permeabilization by SLO and the direct effect of the introduced cytosol itself. In relation to the former factor, we detected an increase in the phosphorylation of p38 MAPK during the permeabilization of HeLa cells as shown in Fig. S4. At present, the precise reason why basal activation of p38 MAPK occurs during permeabilization has not been clarified. In relation to the second factor, western blotting of WT and Db liver lysates indicated that the phosphorylation of p38 MAPK was increased substantially in Db liver (Fig. 4C and 4D, Db). This is consistent with reports that showed the increased phosphorylation of p38 MAPK in the liver of db/db and ob/ob mice, and mice in which obesity was induced by a high fat diet [29], [30], [31]. The authors argued that the increased generation of reactive oxygen species by glycosylation or tumor necrosis factor under diabetic conditions would cause excessive oxidation and cellular stress, and lead to insulin resistance. Activation of p38 MAPK in Db liver might also be attributed to leptin function. Leptin is a hormone that is secreted from adipocytes; it has a major influence on energy balance and has drawn attention owing to its cross-talk with insulin signal transduction. It has been reported that the level of circulating leptin is increased in Db/Db and obese mice [40], [41]. Interestingly, leptin was reported to activate p38 MAPK, which leads to the enhanced expression of collagen and is possibly followed by liver fibrosis [42], [43]. Thus, the activation of p38 MAPK in Db liver or Db cells could be attributed not only to the response to stress but also to the increased level of leptin. In addition, it would be interesting to compare the effect of liver cytosol from the ob/ob mouse or other animal models of diabetes with that of Db cytosol in resealed cells. In particular, given that the ob/ob mouse has a mutation in the leptin gene, we might be able to identify which intracellular events are affected by increased circulating leptin.

In the present study, we found that the substantial activation of p38 MAPK in Db cytosol induced eventually the perturbation of the broad range of early endocytic pathways described below.

### Reconstitution and Analysis of Endocytic Transport in WT and Db Cells

During the process of checking the morphology of organelles and the cytoskeleton in resealed cells by immunofluorescence analysis, we found that the early endosome marker protein EEA1 was associated less with endosomal membranes in Db model cells than in WT model cells (Fig. S2). Provided that EEA1 binds to the early endosomes via PI3P, as is usual [26], [27], we supposed that the amount of PI3P was lower in Db cells than in WT cells. Our FYVE-targeting assay and lipid blot analysis revealed that the amount of PI3P in Db cells was ∼55 % of that in WT cells (Fig. 4B and 4C), which was dependent on p38 MAPK (Fig. 4B). The physiological role of PI3P in endosomes has received much attention with respect to endosomal processing and the transduction of extracellular signals such as EGF and TGFß to the nucleus via endosomes [44]. This is because PI3P in the endosomal membrane seems to act as a microdomain for the recruitment of various proteins that contain a PI3P-binding (FYVE) domain. The functions of FYVE-domain proteins range from endosomal fusion and processing to signal transduction [45], [46], [47]. In addition, depletion of PI3P from early endosomes can modulate the signaling by growth factor receptors such as EGFR by extending or shortening the sojourn time of the receptors in endosomes [44]. Therefore, depletion of PI3P from endosomes under diabetic conditions would have a wide range of influences on the cell, especially in terms of endosomal processing and signal transduction.

We had reported previously that oxidative stress by H_2_O_2_ treatment inhibited a broad range of endocytic pathways [22] including (i) recycling of Tf from endosomes to the plasma membrane, (ii) retrograde transport of cholera toxin from endosomes to the Golgi apparatus, and (iii) the degradation pathway of EGFR. Consequently, we reconstituted early endocytic pathways in WT and Db resealed cells and analyzed at the cellular level the specific perturbation of endocytic processes under pathogenic (diabetic) conditions.

The most important goal of these assays was to detect the various phenotypic differences between WT and Db cytosol. Given that such differences are attributable to different properties of the two types of cytosol, it might be possible to identify the cytosolic factors that perturb the reconstituted reactions in Db cells by analyzing the two cytosols biochemically. In addition, by focusing on only the phenotypic differences between WT and Db cells, we could eliminate the effects of stresses or other artifacts that were induced during permeabilization and resealing.

Unlike in intact HeLa cells treated with H_2_O_2_, we found that internalization of Tf from the plasma membrane and recycling of Tf from endosomes back to the plasma membrane were not inhibited in Db cells and were indistinguishable from those processes in WT cells (Fig. S3). Furthermore, the retrograde transport of Ctx from endosomes to the Golgi apparatus was delayed in Db cells as compared with WT cells, and unlike the recycling of Tf, the delay in Ctx transport was restored in the presence of a specific inhibitor of p38 MAPK, SB203580 and SB202190 (Fig. 6B and 6C). These findings suggested that the retrograde transport of Ctx was regulated by the amount of PI3P in early endosomes in a p38 MAPK-dependent manner. These results were consistent with our previous report [22], and confirmed that retrograde transport was reconstituted successfully in resealed cells.

Even in the absence of EGF, internalization of some EGFR from the cell surface to the endosomes was observed in both WT and Db cells (Fig. 7A, WT and Db). The internalization was inhibited completely by the p38 MAPK inhibitor SB203580 (Fig. 7A, WT+SB and Db+SB). We believed that, as described above, cytosol prepared from mouse liver (WT or Db cytosol) had a basal level of activated p38 MAPK, which induced the internalization of EGFR even in the absence of EGF stimulation. In fact, Vergarajauregui et al. [48] and Adachi et al. [49] have reported that the activation of p38 MAPK induces the internalization and degradation of EGFR without EGF stimulation through p38 MAPK phosphorylation of Ser and Thr residues in EGFR.

Interestingly, upon EGF stimulation, the degradation of EGFR was enhanced in Db cells as compared with WT cells (Fig. 7B and C, WT and Db), and the increase in EGFR degradation was not affected by the p38 MAPK inhibitor SB203580 (Fig. 7B and C, WT+SB and Db+SB). EGFR-mediated signal transduction might be perturbed under diabetic conditions in hepatocytes through activated p38 MAPK inducing the enhancement of EGFR internalization and inhibition of Erk phosphorylation through direct interaction. This is conceivable because Zhang et al. [50] demonstrated that p38 MAPK interacts directly with Erk and inhibits Erk phosphorylation. However, this is unlikely to be the case because the enhanced degradation of EGFR in Db cells was independent of p38 MAPK (Fig. 7B and C, WT+SB and Db+SB). Thus, although the precise mechanism remains unclear, the degradation of EGFR in Db cells upon EGF stimulation apparently occurred in a p38 MAPK-independent manner.

### Possible Future Application of Disease Model Cell System Using Cell-resealing Technique

In this study, we observed the cytosol dependent-depletion of PI3P from endosomes, inhibition of Ctx transport to the Golgi, and enhanced EGFR degradation in Db cells. The detection of these dysfunctions in diabetic liver or in isolated diabetic hepatocytes would be required to validate the model system. In fact, our finding of the enhanced degradation of EGFR in Db cells seems to be one example. Several studies have also shown that the number of surface EGFR molecules is decreased in hepatocytes isolated from the streptozotocin-induced diabetes rat model [51]–[54]. If the enhanced degradation of EGFR occurs under diabetic conditions in hepatocytes, this could explain the decrease in the EGFR receptor at the cell surface in such cells. The next step is to investigate the physiological relevance of the enhanced degradation using the resealed cell system or intact hepatoma cells. In addition, validation of the decreased level of PI3P and the inhibition of Ctx transport in diabetic hepatocytes should be performed in future studies.

The disease model cells enabled us to perform various cell-based assays in cells under disease conditions, and should be helpful for elucidating which intracellular events are perturbed at the cellular level under pathogenic conditions. Furthermore, biochemical manipulation of the cytosol, for example by immunodepletion or the addition of function-blocking antibodies, should enable key pathological factors to be identified. Changing the intracellular environment to a disease state using the cell resealing technique should provide a unique and powerful tool to create disease model cells and understand the aberrant processes that occur under pathogenic conditions.

## Materials and Methods

### Reagents and Antibodies

GTP, ATP, creatine phosphate, and creatine kinase were obtained from Sigma. PI was purchased from Molecular Probes. SB203580 and SB202190 were purchased from Calbiochem. Dextran (3, 10, 40, 75, and 2000 kDa) conjugated with fluorescein, tetramethylrhodamine (TMR), or Alexa647 was purchased from Invitrogen. Other reagents were purchased from Wako Chemicals. The following primary antibodies were used: rabbit anti-EGFR antibody (Cell Signaling Technology); rabbit anti-COX IV antibody (Cell Signaling Technology); rabbit anti-p38 antibody (Cell Signaling Technology); rabbit anti-phospho-p38 MAPK (Thr180/Tyr182) antibody (Cell Signaling Technology); mouse anti-GM130 antibody (BD Transduction Laboratories); Alexa 488-conjugated anti-GST antibody (Molecular Probes); mouse anti-GST antibody (Cell Signaling Technology). The following secondary antibodies were used: HRP-conjugated anti-mouse or anti-rabbit (Chemicon) antibodies; Cy2-conjugated anti-mouse or anti-rabbit antibodies (Chemicon); Cy3-conjugated anti-mouse or anti-rabbit antibodies (Chemicon).

### Preparation of Semi-intact Cells

HeLa cells were grown on glass-bottomed dishes (IWAKI). The cells were washed twice with PBS and then incubated with streptolysin O (SLO; Bioacademia), at the concentrations indicated in the text, on ice for 5 min. After washing three times with PBS, the cells were incubated with transport buffer (TB: 25 mM HEPES-KOH, pH 7.4, 115 mM potassium acetate, 2.5 mM MgCl_2_) that contained 300 µg/ml PI (Molecular Probes) at 32°C for 5 min. The cells were washed twice with TB, and were treated with 1 µg/ml Hoechst 33342 (Dojindo) at room temperature for 10 min to stain the nucleus. The cells were observed with an LSM710 confocal microscope (Carl Zeiss). We counted the number of cells that were positive for staining with Hoechst only (N_[H]_) or Hoechst and PI (N_[H+P]_). We performed three independent experiments, and counted 300 cells in each experiment. The means and standard deviations of the percentage N_[H+P]_/N_[H+P]_ + N_[H]_ are shown in the graphs.

### Resealing of Semi-intact Cells

Cytosol was prepared from murine lymphoma L5178Y cells as described in [55]. Semi-intact cells were incubated with L5178Y cytosol and an ATP regenerating system (1 mM ATP, 8 mM creatine kinase, and 50 µg/ml creatine phosphate), 1 mg/ml glucose, 1 mM GTP, 100 µg/ml fluorescently-labeled dextran (3, 10, 45, 70, 2000 kDa, Invitrogen) at 32°C for 15 min. CaCl_2_ was added at the concentrations indicated in the text and the cells were incubated at 32°C for a further 5 min. The cells were then washed twice with PBS and were further incubated with prewarmed DMEM supplemented with 10% FCS at 37°C in 5% CO_2_ for 30 min. The cells were observed by confocal microscopy. We counted the number of cells that were positive for staining with PI only (N_[P]_) or PI and dextran (N_[P+D]_). We performed three independent experiments, and counted 300 cells in each experiment. The means and standard deviations of the percentage N_[P+D]_/ N_[P+D]_ +N_[P]_ are shown in the graphs.

### Preparation of Cytosol

Cytosol from L5178Y cells was prepared as described in [55]. Cytosol was prepared from the liver of WT or db/db mice (CLEA Japan), and the experiments using mice were conducted with the approval of the animal experiment ethics committee at Graduate School of Arts and Sciences, the University of Tokyo, and according to the guidelines for the care and use of laboratory animals. Livers were isolated from WT or db/db mice and were minced finely with scissors in homogenization buffer (10 mM Hepes pH 7.4, 0.25 M sucrose, 1 mM EDTA, 0.5 mM DTT, 0.2 mM PMSF) on ice. The minced livers were washed three times with homogenization buffer by decantation, and homogenized with three volumes of homogenization buffer using a loosely fitting Potter homogenizer (3 to 5 strokes). The homogenate was centrifuged at 9500 rpm (MX-300 microcentrifuge; TOMY) for 10 min at 4°C to remove cellular debris. The supernatant was centrifuged at 9500 rpm for a further 30 min at 4°C, followed by centrifugation at 15000 rpm for 30 min at 4°C. The supernatant was then ultracentrifuged at 65000 rpm at 4°C in an Optima TLX ultracentrifuge (Beckman) using a TLX100 rotor. The supernatant was dialyzed in TB for 4 hr at 4°C, and then stored in liquid nitrogen.

### Flow Cytometry

HeLa cells were incubated with SLO on ice for 5 min, and then were incubated further with transport buffer (TB) containing propidium iodide (PI) at 32°C for 5 min. Semi-intact HeLa cells were incubated with 100 µg/ml of 3, 10, 40, 70, or 2000 kDa dextran conjugated with fluorescein in the presence of 1.5 mg/ml L5178Y cytosol and ATP/GTP/glucose at 32°C for 15 min. After resealing with 1 mM CaCl_2_ at 32°C for 5 min, the cells were further incubated with prewarmed DMEM supplemented with 10% FCS at 37°C in 5% CO_2_ for 30 min. the cells were trypsinized and subjected to flow cytometry using the Guava easyCyte 8 HT flow cytometry system.

### Conventional Electron Microscopy

HeLa cells were cultured on plastic coverslips (LF1, Sumitomo Bakelite). The cells were permeabilized with SLO and were incubated with 3 mg/ml WT or Db liver cytosol that contained an ATP regenerating system, 1 mg/ml glucose, 1 mM GTP, at 32°C for 30 min. The cells were resealed by the addition of 1 mM CaCl_2_ at 32°C for 5 min and then were further incubated with DMEM with FCS at 37°C in 5% CO_2_ for 30 min. The cells were fixed in 2.5% glutaraldehyde (Electron Microscopy Sciences) in 0.1 M sodium phosphate buffer, pH 7.4 (PB), for 2 h. After washing with PB, the specimens were post-fixed in 1% OsO_4_ in PB for 60 min at room temperature, and washed with distilled water. The specimens were dehydrated in a series of graded ethanol solutions and embedded in epoxy resin. Ultra-thin sections were doubly stained with uranyl acetate and lead citrate and observed under an H7600 electron microscope (Hitachi).

### GST-2xFYVE Targeting Assay

A recombinant tandem FYVE domain from mouse Hrs was produced as a glutathione S-transferase (GST) fusion protein (GST-2xFYVE) as described in [22]. The GST-2xFYVE targeting assay was performed as described in [22] with slight modification. Briefly, semi-intact HeLa cells were incubated with 3 mg/ml WT or Db liver cytosol that contained an ATP regenerating system, 1 mg/ml glucose, and 1 mM GTP at 32 °C for 30 min. Then, 1 µg of GST-2xFYVE protein was added to the 100 µl reaction mixture, and the cells were further incubated at 32°C for 15 min. The cells were washed with TB, fixed with 3% paraformaldehyde for 20 min, and then permeabilized with 0.2% Triton X-100 for 15 min. After blocking with 3% skim milk, GST-2xFYVE was visualized with the Alexa 488-conjugated anti-GST antibody. The cells were observed and Z-stack images were taken every 1.5 µm with an LSM710 confocal microscope (Carl Zeiss). The mean fluorescence intensity in control cells was assigned as 100%.

### Lipid Blot Analysis Using GST-2xFYVE Proteins

Lipid blot analysis was performed as described in previously [22] with slight modification. Briefly, acidic lipids were extracted from resealed WT or Db cells as described in [22]. Dried lipid samples were reconstituted with 5 µl of CHCl_3_:MeOH:H_2_O (1∶2∶0.8). The samples were vortexed for 30 s, and then sonicated in an ice water bath for 5 min. The samples were vortexed again for 30 s and spotted onto a nitrocellurose membrane. After drying at room temperature, the membrane was blocked with 3% bovine serum albumin (BSA) in PBST (BSA-PBST) for 1 hr at room temperature. We then incubated the membrane with 10 µg/ml GST-2xFYVE proteins in BSA-PBST for 3 hr at room temperature. After washing with PBST for 5 min three times, the membrane was incubated with anti-GST antibody for 2 hr at room temperature. The membrane was then washed with PBST for 5 min three times and incubated with anti-mouse IgG-HRP for 1 hr. After wash with PBST three times, PI3P was detected by enhanced chemiluminescence (Western Lightning Plus-ECL, PerkinElmer). We quantified the intensity of the dots in each sample and assigned the value of the content of PI3P in WT cells to be 100% to allow comparison among experimental samples. The experiments were performed in triplicate, and the means and standard deviations are shown in the graph.

### Western Blot

Cell lysates were separated by SDS-PAGE, and were transferred to nitrocellulose membrane using a semi-dry blotting system (Bio-Rad). For the Western blot of EGFR, proteins were transferred onto the nitrocellulose membrane using a semi-dry blotting apparatus at 2 mA/cm^2^ for 30 min according to the method of Kyhse-Andersen [56]. After blocking with 3% BSA or skim milk in TBS, the membrane was incubated with primary antibody at room temperature for 2 hr or at 4°C for over night, and then further with HRP-conjugated secondary antibody at room temperature at 1 hr. Then the protein was detected by enhanced chemiluminescence (Western Lightning Plus-ECL, PerkinElmer).

### Indirect Immunofluorescence Method

The cells were fixed with 3% paraformaldehyde for 20 min, and then permeabilized with 0.2% Triton X-100 in PBS for 15 min at room temperature. After blocking with 3% BSA or 5% skim milk for 30 min, the cells were incubated with appropriate primary antibodies for 2 h. This was followed by incubation with the appropriate secondary antibody for 1 h. The cells were observed by using an LSM710 confocal microscope with a 63× Plan-Neofluar oil immersion objective (NA  =  1.4). For the images of microtubules, Z stack images were obtained and were stacked to show the complete array of microtubules.

### Endocytosis Assay for Alexa 546-labeled Cholera Toxin B Subunit (CtxB)

Semi-intact HeLa cells were incubated with WT or Db cytosol in the presence of ATP/GTP/glucose and 100 µg/ml Alexa647-conjugated dextran for 30 min, and then resealed with 1 mM CaCl_2_ for 5 min. Resealed HeLa cells that contained WT or Db liver cytosol were placed on ice for 5 min. The cells were incubated with 300 µg/ml Alexa 546-CtxB (Molecular Probes) on ice for 30 min. After washing three times with PBS, the cells were incubated with pre-warmed DMEM supplemented with 10% FCS at 37 °C for the indicated times. At the indicated times after incubation with pre-warmed DMEM supplemented with 10% FCS at 37°C to initiate the transport of CtxB, WT or Db cells were fixed and immunostained with antibody against GM130, a cis-Golgi marker, and were observed by an LSM710 confocal microscope (Carl Zeiss). We could distinguish the resealed cells from non-resealed cells by the labeling of resealed cells with Alexa647-conjugated dextran, and compared the difference in Ctx transport between WT and Db cells. We counted the number of cells in which Ctx was colocalized with GM130, in triplicate (300 cells were counted for each experiment). The means and the standard deviations for the number of cells in which CtxB was colocalized with GM130 are shown as a graph. The results were verified by applying Student’s t-test.

### EGFR Degradation Assay

HeLa cells were incubated with DMEM without FCS overnight. HeLa cells that had been grown on a cover slip were permeabilized with SLO and incubated with 3 mg/ml WT or Db liver cytosol that contained an ATP regenerating system, 1 mg/ml glucose, 1 mM GTP, and 100 µg/ml TMR-conjugated dextran at 32°C for 30 min. The cells were resealed by the addition of 1 mM CaCl_2_ at 32°C for 5 min. After incubating with DMEM without FCS at 37°C in 5% CO_2_ for 30 min, the cells were treated with 10 ng/ml EGF (PeproTech) in DMEM without FCS at 37°C for the indicated times. The cells were fixed for indirect immunofluorescence analysis using antibodies against EGFR or were lysed for western blotting using antibodies against EGFR and COX IV. The intensities of the EGFR and COX IV bands were quantified, and we assigned the value of intensity [EGFR]/ intensity [COX IV] at the 0 min time point to be 100%. The means and standard deviations are plotted in the graph.

### Statistical Analysis

Data analysis was carried out using the F-test to check the equality of variance and then Student’s or Welch’s *t* test. In Fig. 5B and 6C, statistical analysis was performed using one-way ANOVA and Dunnett’s post hoc test. Experiments were performed at least three times. Values are expressed as the mean ± standard deviation (SD), and data were considered significant at *P < 0.05, ** *P* < 0.01.

## Supporting Information

Figure S1 **Morphology of organelles and the cytoskeleton** **in intact and resealed cells.** Semi-intact HeLa cells were incubated with 1.5 mg/ml L5178Y cytosol, an ATP regenerating system, GTP, glucose, and fluorescein-dextran at 32°C for 15 min, and then resealed by treatment with 1 mM CaCl_2_ at 32°C for 5 min. The cells were incubated with DMEM supplemented with FCS at 37°C for 30 min. Intact and resealed HeLa cells were stained with antibodies against GM130, ERGIC53, EEA1, Lamp1, cytochrome C, actin, tubulin, or vimentin, or with ER tracker and Hoechst 333342. The information about antibodies and reagents is described in Materials S1. Bar = 10 µm.
(TIF)

Figure S2 **Morphology of organelles and the cytoskeleton in WT and Db cells.** Semi-intact HeLa cells were incubated with 3 mg/ml WT or Db liver cytosol, an ATP regenerating system, GTP, glucose, and fluorescein-dextran at 32°C for 30 min, and then resealed by treatment with 1 mM CaCl_2_ at 32°C for 5 min. The cells were incubated with DMEM supplemented with FCS at 37°C for 30 min. WT and Db cells were stained with antibodies against GM130, ERGIC53, EEA1, Lamp1, cytochrome C, actin, tubulin, or vimentin, or with ER tracker and Hoechst 333342. The information about antibodies and reagents is described in Materials S1. Bar = 10 µm.
(TIF)

Figure S3 **Recycling of transferrin in WT and Db cells.** A. HeLa cells that had been grown on a cover slip were permeabilized with SLO and incubated with 3 mg/ml WT or Db liver cytosol that contained an ATP regenerating system, 1 mg/ml glucose, 1 mM GTP, and 100 µg/ml TMR-conjugated dextran at 32°C for 30 min. The cells were resealed by the addition of 1 mM CaCl_2_ at 32°C for 5 min. After incubating with DMEM without FCS at 37°C in 5% CO_2_ for 30 min, the cells were incubated on ice for 5 min, and then for a further 30 min on ice with 10 µg/ml Alexa 488-conjugated Tf (Molecular Probes) in DMEM (without FCS). After washing twice with PBS, the cells were incubated with DMEM containing 10% FCS and unlabeled Tf at 37°C for the indicated times. The cells were fixed and observed with an LSM710 confocal microscope (Carl Zeiss). At 0 min, images just under the cell surface were obtained with a confocal microscope to show that almost all the transferrin remained at the plasma membrane at 0 min, and no endocytosed transferrin was observed. Bar  =  10 µm. B. The fluorescence intensity of Alexa488-Tf that was bound to the plasma membrane of WT and Db cells was measured, and the means and standard deviations for % fluorescence intensity are shown in the graph. C. The fluorescence intensity of Alexa488-Tf in WT and Db cells was measured after a 10 or 20 min chase by taking the Z-stack images every 1.5 µm and measuring the mean fluorescence intensity. The means and standard deviations for the % fluorescence intensity are shown in the graph. D. Resealed WT or Db cells were prepared as described in A. The cells were incubated with 10 µg/ml Tf conjugated with Alexa Fluor 488 (Molecular Probes) on ice for 30 min, and then with medium at 37°C for 1, 10, and 20 min. After the Alexa 488-conjugated Tf had been removed by washing the cells with acidic wash buffer (DMEM, pH 4.0), the cells were trypsinized, resuspended, and subjected to flow cytometry using a Guava easyCyte 8 HT flow cytometry system. E. WT and Db cells were treated as described in D, and the means and standard deviations for the fluorescence intensity of Alexa488-Tf are shown in the graph. Three independent experiments were performed.
(TIF)

Figure S4 **Phosphorylation status of p38 MAPK, p42/44 MAPK, and JNK in resealed cells.** Semi-intact HeLa cells were incubated with 1.5 mg/ml L5178Y cytosol, an ATP regenerating system, GTP, and glucose at 32°C for 15 min, and then resealed by treatment with 1 mM CaCl_2_ at 32°C for 5 min. The cells were incubated with DMEM supplemented with FCS at 37°C for 0, 30, 60, 90, and 120 min. Intact or the resealed HeLa cells were lysed and were subjected to western blotting using antibodies against p38 MAPK, phospho-p38 MAPK, p42/44 MAPK, phospho-p42/44 MAPK, JNK, and phospho-JNK. The information about antibodies is described in Materials S1.
(TIF)

Materials S1 **The list of antibodies and reagents used in Figure S1, S2, and S4.**
(DOCX)
