# Supplementary figures and images for: A Resealed-Cell System for Analyzing Pathogenic Intracellular Events: Perturbation of Endocytic Pathways under Diabetic Conditions
Source: PLoS One. 2012 Aug 29;7(8):e44127. doi: 10.1371/journal.pone.0044127 (PMC3430665; doi:10.1371/journal.pone.0044127)

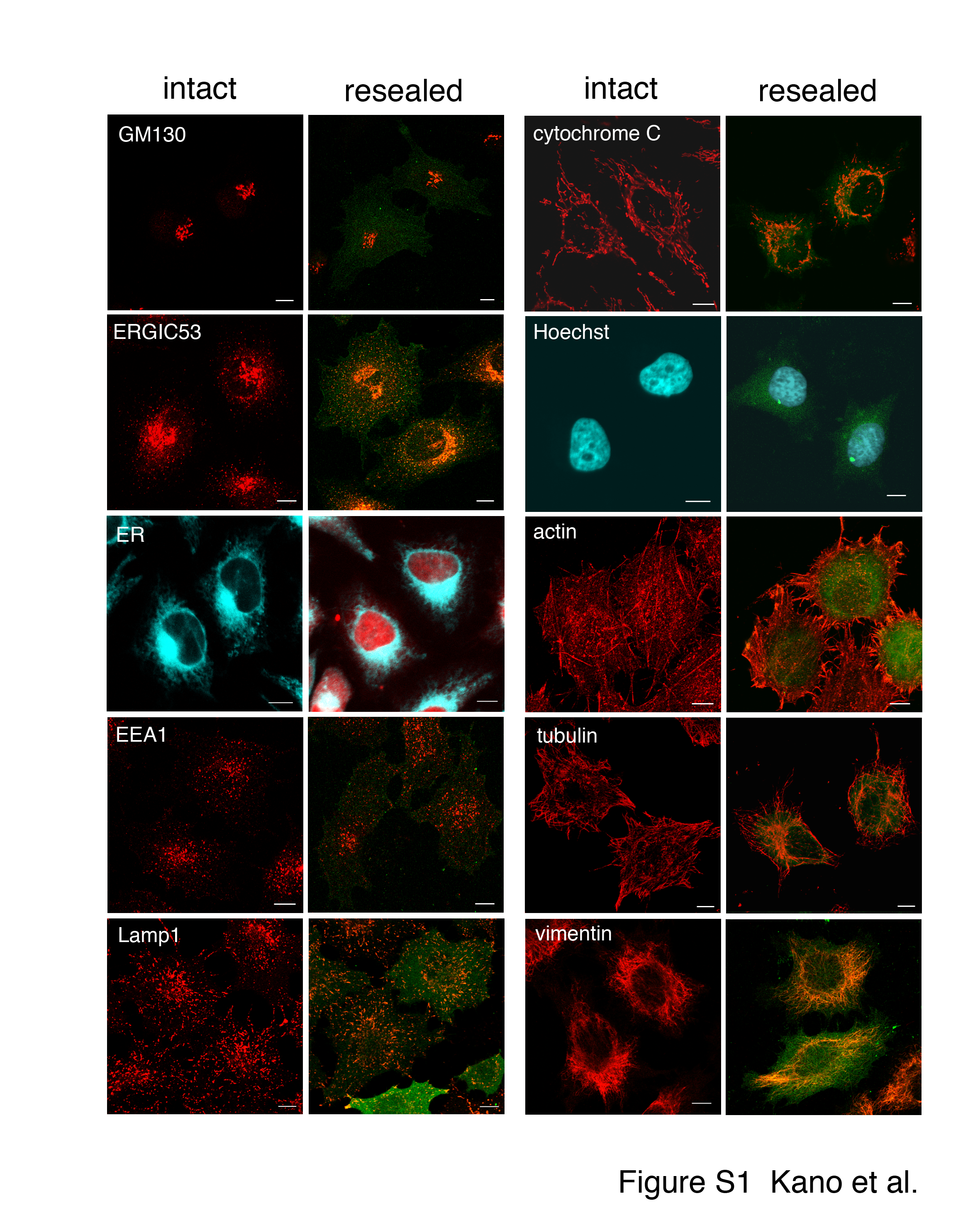

Supplement: Figure S1 — Morphology of organelles and the cytoskeleton in intact and resealed cells. Semi-intact HeLa cells were incubated with 1.5 mg/ml L5178Y cytosol, an ATP regenerating system, GTP, glucose, and fluorescein-dextran at 32°C for 15 min, and then resealed by treatment with 1 mM CaCl2 at 32°C for 5 min. The cells were incubated with DMEM supplemented with FCS at 37°C for 30 min. Intact and resealed HeLa cells were stained with antibodies against GM130, ERGIC53, EEA1, Lamp1, cytochrome C, actin, tubulin, or vimentin, or with ER tracker and Hoechst 333342. The information about antibodies and reagents is described in Materials S1. Bar = 10 µm. (TIF) [file pone.0044127.s001.tif]

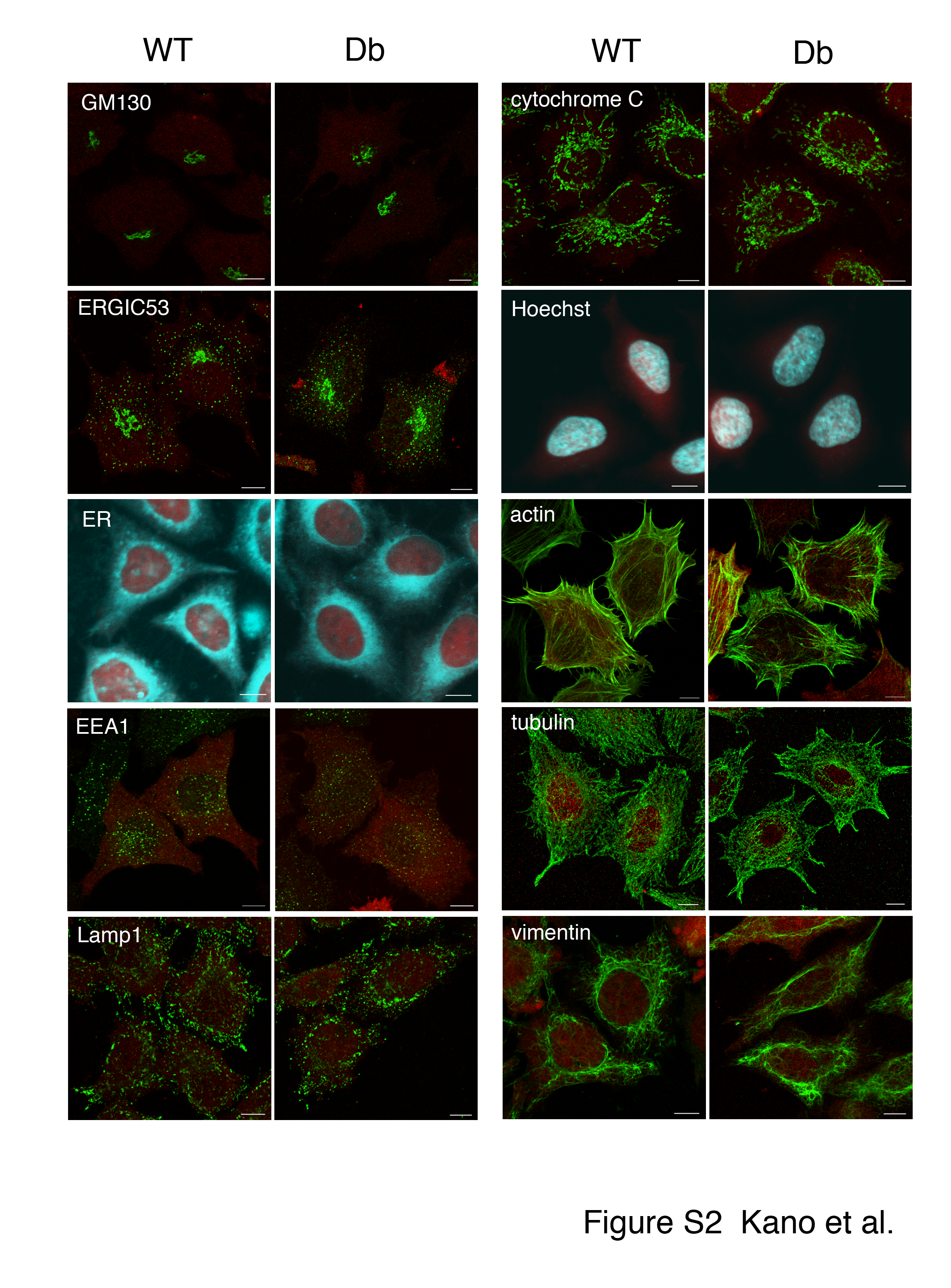

Supplement: Figure S2 — Morphology of organelles and the cytoskeleton in WT and Db cells. Semi-intact HeLa cells were incubated with 3 mg/ml WT or Db liver cytosol, an ATP regenerating system, GTP, glucose, and fluorescein-dextran at 32°C for 30 min, and then resealed by treatment with 1 mM CaCl2 at 32°C for 5 min. The cells were incubated with DMEM supplemented with FCS at 37°C for 30 min. WT and Db cells were stained with antibodies against GM130, ERGIC53, EEA1, Lamp1, cytochrome C, actin, tubulin, or vimentin, or with ER tracker and Hoechst 333342. The information about antibodies and reagents is described in Materials S1. Bar = 10 µm. (TIF) [file pone.0044127.s002.tif]

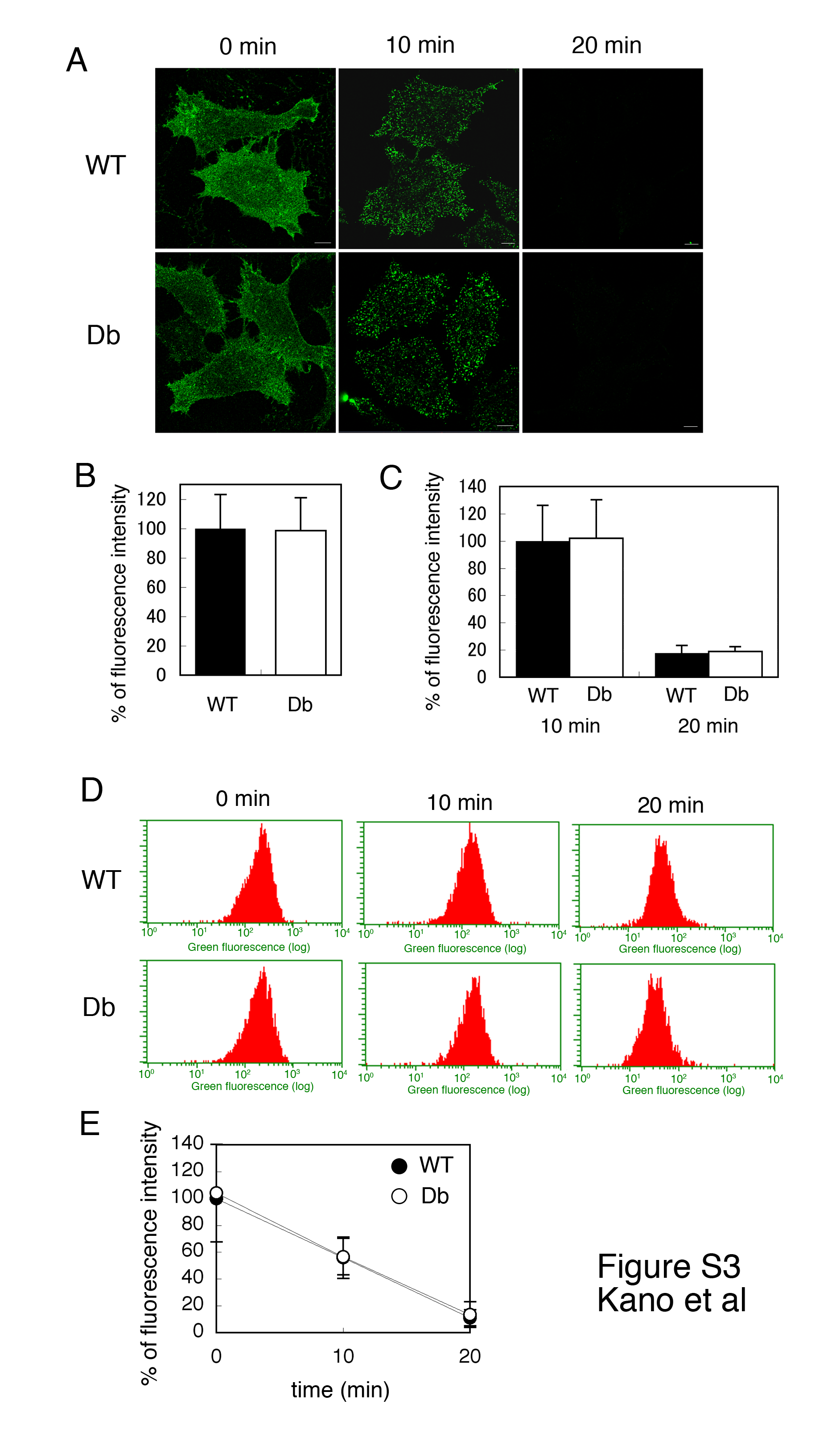

Supplement: Figure S3 — Recycling of transferrin in WT and Db cells. A. HeLa cells that had been grown on a cover slip were permeabilized with SLO and incubated with 3 mg/ml WT or Db liver cytosol that contained an ATP regenerating system, 1 mg/ml glucose, 1 mM GTP, and 100 µg/ml TMR-conjugated dextran at 32°C for 30 min. The cells were resealed by the addition of 1 mM CaCl2 at 32°C for 5 min. After incubating with DMEM without FCS at 37°C in 5% CO2 for 30 min, the cells were incubated on ice for 5 min, and then for a further 30 min on ice with 10 µg/ml Alexa 488-conjugated Tf (Molecular Probes) in DMEM (without FCS). After washing twice with PBS, the cells were incubated with DMEM containing 10% FCS and unlabeled Tf at 37°C for the indicated times. The cells were fixed and observed with an LSM710 confocal microscope (Carl Zeiss). At 0 min, images just under the cell surface were obtained with a confocal microscope to show that almost all the transferrin remained at the plasma membrane at 0 min, and no endocytosed transferrin was observed. Bar = 10 µm. B. The fluorescence intensity of Alexa488-Tf that was bound to the plasma membrane of WT and Db cells was measured, and the means and standard deviations for % fluorescence intensity are shown in the graph. C. The fluorescence intensity of Alexa488-Tf in WT and Db cells was measured after a 10 or 20 min chase by taking the Z-stack images every 1.5 µm and measuring the mean fluorescence intensity. The means and standard deviations for the % fluorescence intensity are shown in the graph. D. Resealed WT or Db cells were prepared as described in A. The cells were incubated with 10 µg/ml Tf conjugated with Alexa Fluor 488 (Molecular Probes) on ice for 30 min, and then with medium at 37°C for 1, 10, and 20 min. After the Alexa 488-conjugated Tf had been removed by washing the cells with acidic wash buffer (DMEM, pH 4.0), the cells were trypsinized, resuspended, and subjected to flow cytometry using a Guava easyCyte 8 HT flow cytometry system. E. W [file pone.0044127.s003.tif]

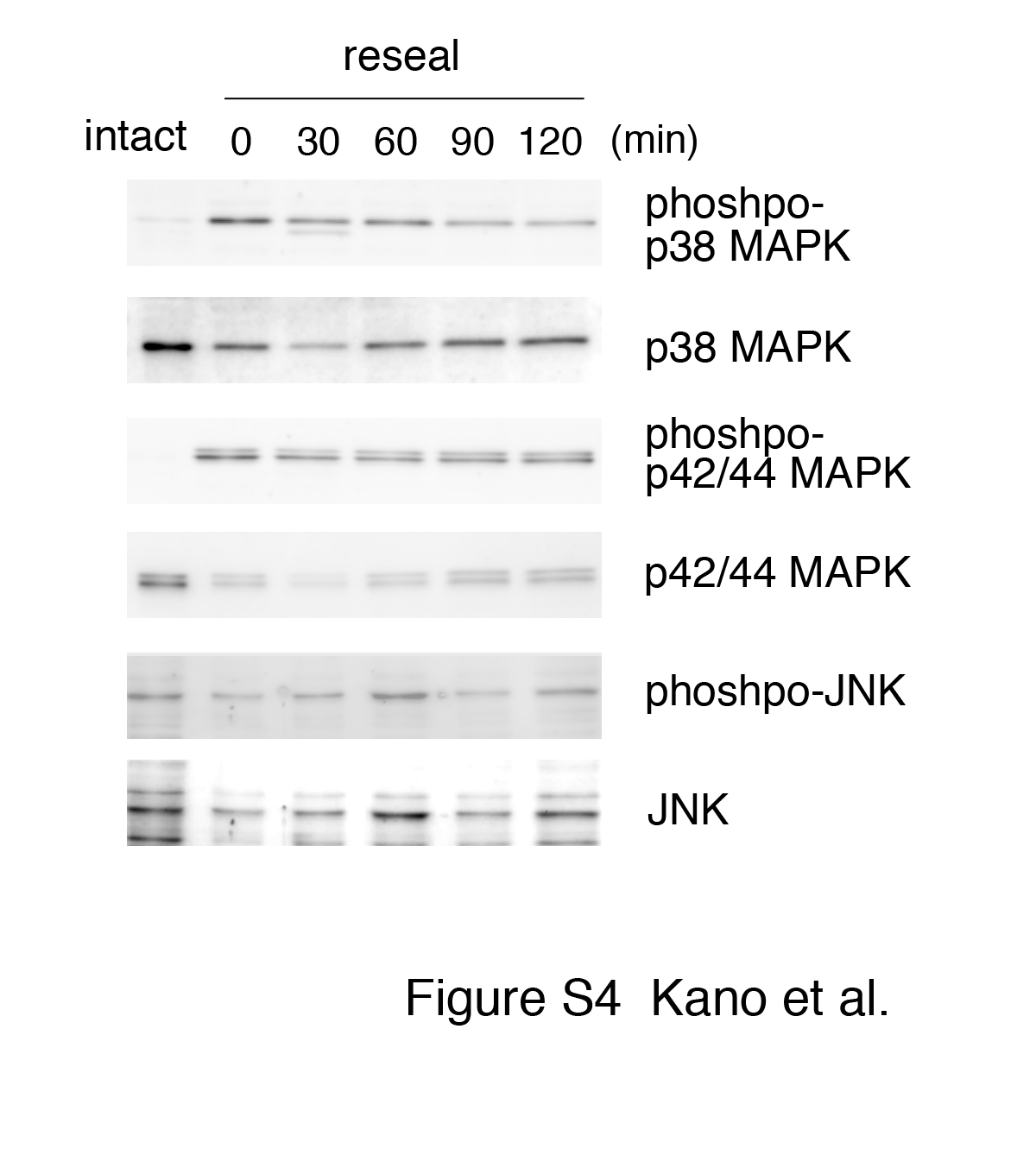

Supplement: Figure S4 — Phosphorylation status of p38 MAPK, p42/44 MAPK, and JNK in resealed cells. Semi-intact HeLa cells were incubated with 1.5 mg/ml L5178Y cytosol, an ATP regenerating system, GTP, and glucose at 32°C for 15 min, and then resealed by treatment with 1 mM CaCl2 at 32°C for 5 min. The cells were incubated with DMEM supplemented with FCS at 37°C for 0, 30, 60, 90, and 120 min. Intact or the resealed HeLa cells were lysed and were subjected to western blotting using antibodies against p38 MAPK, phospho-p38 MAPK, p42/44 MAPK, phospho-p42/44 MAPK, JNK, and phospho-JNK. The information about antibodies is described in Materials S1. (TIF) [file pone.0044127.s004.tif]
