# Supplementary material for: A Resealed-Cell System for Analyzing Pathogenic Intracellular Events: Perturbation of Endocytic Pathways under Diabetic Conditions
Source: PLoS One. 2012 Aug 29;7(8):e44127. doi: 10.1371/journal.pone.0044127 (PMC3430665; doi:10.1371/journal.pone.0044127)
Supplement: Materials S1 — The list of antibodies and reagents used in Figure S1, S2, and S4. (DOCX) [file pone.0044127.s005.docx]

**Materials S1. The list of antibodies and reagents used in Figure S1, S2, and S4.**

The following primary antibodies were used: rabbit anti-EEA1 antibody (Acris Antibodies); rabbit anti-ERGIC53 antibody (Sigma); mouse anti-GM130 antibody (BD Transduction Laboratories); mouse anti-Lamp1 antibody (H4A3, Developmental Studies Hybridoma Bank, The University of Iowa); mouse anti-cytochrome C antibody (BD Pharmingen); rabbit anti-ß−actin antibody (Sigma); rabbit anti- ß-tubulin antibody (Sigma); mouse anti-vimentin antibody (Sigma); rabbit anti-p42/44 MAPK antibody (Cell Signaling Technology); rabbit anti-phospho-p42/44 MAPK (Thr202/Tyr204) antibody (Cell Signaling Technology); rabbit anti-JNK/SAPK antibody (Cell Signaling Technology); rabbit anti-phoshpo-JNK/SAPK (Thr183/Tyr185) antibody (Cell Signaling Technology). ER tracker Blue-White DPX was purchased from Invitrogen.
